# An agent-based model of binge drinking, inequitable gender norms and their contribution to HIV transmission, with application to South Africa

**DOI:** 10.1186/s12879-023-08470-y

**Published:** 2023-07-29

**Authors:** Leigh F. Johnson, Mmamapudi Kubjane, Alex de Voux, Julius Ohrnberger, Mpho Tlali

**Affiliations:** 1 Centre for Infectious Disease Epidemiology and Research, Faculty of Health Sciences, University of Cape Town, Anzio Road, Cape Town, 7925 Observatory South Africa; 2 Division of Epidemiology and Biostatistics, University of Cape Town, Cape Town, South Africa; 3 Department of Infectious Disease Epidemiology, Imperial College London, London, UK

**Keywords:** HIV/AIDS, Structural intervention, Alcohol, Gender norms, Mathematical modelling, South Africa

## Abstract

**Background:**

Binge drinking, inequitable gender norms and sexual risk behaviour are closely interlinked. This study aims to model the potential effect of alcohol counselling interventions (in men and women) and gender-transformative interventions (in men) as strategies to reduce HIV transmission.

**Methods:**

We developed an agent-based model of HIV and other sexually transmitted infections, allowing for effects of binge drinking on sexual risk behaviour, and effects of inequitable gender norms (in men) on sexual risk behaviour and binge drinking. The model was applied to South Africa and was calibrated using data from randomized controlled trials of alcohol counselling interventions (*n* = 9) and gender-transformative interventions (*n* = 4) in sub-Saharan Africa. The model was also calibrated to South African data on alcohol consumption and acceptance of inequitable gender norms. Binge drinking was defined as five or more drinks on a single day, in the last month.

**Results:**

Binge drinking is estimated to be highly prevalent in South Africa (54% in men and 35% in women, in 2021), and over the 2000–2021 period 54% (95% CI: 34–74%) of new HIV infections occurred in binge drinkers. Binge drinking accounted for 6.8% of new HIV infections (0.0–32.1%) over the same period, which was mediated mainly by an effect of binge drinking in women on engaging in casual sex. Inequitable gender norms accounted for 17.5% of incident HIV infections (0.0–68.3%), which was mediated mainly by an effect of inequitable gender norms on male partner concurrency. A multi-session alcohol counselling intervention that reaches all binge drinkers would reduce HIV incidence by 1.2% (0.0–2.5%) over a 5-year period, while a community-based gender-transformative intervention would reduce incidence by 3.2% (0.8–7.2%) or by 7.3% (0.6–21.2%) if there was no waning of intervention impact.

**Conclusions:**

Although binge drinking and inequitable gender norms contribute substantially to HIV transmission in South Africa, recently-trialled alcohol counselling and gender-transformative interventions are likely to have only modest effects on HIV incidence. Further innovation in developing locally-relevant interventions to address binge drinking and inequitable gender norms is needed.

**Supplementary Information:**

The online version contains supplementary material available at 10.1186/s12879-023-08470-y.

## Introduction

Binge drinking, inequitable gender norms and sexual risk behaviour are closely interlinked. Binge drinking is strongly associated with transactional, casual and condomless sex [1–10] and multiple and concurrent partnerships [9–13]. Inequitable gender norms are associated with male partner concurrency [14–18], unprotected sex [10, 19, 20], binge drinking [15–17, 21] and male perpetration of intimate partner violence (IPV) [15, 16]. Indeed, binge drinking, IPV and HIV risk behaviour are often described as forming a ‘Substance Abuse, Violence and AIDS’ (SAVA) syndemic [17, 22]. Various interventions to address these structural drivers of HIV risk behaviour have been tested. These include individual-level alcohol counselling interventions, which usually target people at a high risk of alcohol misuse/dependence [23, 24], and gender-transformative interventions, which aim to change attitudes towards gender norms, either through community-level or individual-level interventions [25].

South Africa is particularly challenged by this syndemic. It has one of the highest levels of binge drinking in sub-Saharan Africa, being second only to Eswatini and Namibia in per-capita levels of alcohol consumption among drinkers [26]. It also has relatively high levels of gender-based violence [27], and it has the largest HIV epidemic in the world [28]. Although South African HIV programmes have been successful in reducing HIV incidence, adult HIV incidence rates remain high [29]. South Africa’s HIV response has focused largely on biomedical interventions, with relatively little attention being paid to structural drivers [30].

Randomized controlled trials of structural interventions have had mixed success. Results of these trials are difficult to synthesize, because the interventions that are tested are often heterogeneous and target different populations. In addition, because these are structural interventions, their impact depends on the salience of the associated structural drivers in the local context [31]. There are often multiple endpoints: typically, the structural driver itself, more proximal behavioural factors, and biological endpoints such as HIV, other sexually transmitted infections (STIs) and/or pregnancy. The multiplicity of intervention designs, social contexts and endpoints makes meta-analysis challenging. However, agent-based models (ABMs) may provide a useful framework for synthesizing and extrapolating from the available data on structural interventions [32, 33]. ABMs can be used to simulate individuals, their environments and behaviours, and the health outcomes associated with those environments and behaviours [34], allowing us to test the multiple effects of interventions in a virtual world that is calibrated to match real-world data. Although a number of previous ABMs have evaluated the impact of alcohol counselling interventions [35, 36], there have been no similar evaluations of gender-transformative interventions, and there have been few attempts to reconcile effect measures for different trial endpoints.

We aim to demonstrate how ABMs can be used to simulate the impact of structural drivers on multiple outcomes, and how these models can be calibrated to RCT data, with application to alcohol counselling and gender transformative interventions. We apply the model to South Africa, as a case study of a setting in which alcohol and gender inequality are believed to be major drivers of HIV. We also aim to demonstrate how the same model can be used to assess structural intervention impacts at a population level, and to isolate the causal pathways that are most critical to intervention impact.

## Methods

### Model structure

We used an established ABM, Microsimulation for the Control of South African Morbidity and Mortality (MicroCOSM) [37]. The model simulates a nationally representative sample of 20 000 South Africans, starting in 1985, and tracks changes in the population as a result of birth and death. Each individual is assigned demographic variables (age, sex, race), socio-economic variables (educational attainment, urban/rural location, migration and incarceration history), healthcare access variables (contraceptive use, condom use, HIV testing history, male circumcision, antiretroviral treatment [ART] use), sexual behaviour variables (sexual experience, sexual preference, marital status, risk group [propensity for concurrent partners], number of current partners, commercial sex activity) and health outcomes (HIV, other STIs, parity and mortality). A more detailed description of the model is available elsewhere [37].

For the purpose of the current study, a number of extensions were made to the model. Each individual is randomly assigned a conscientiousness score (defined in terms of the Five Factor Model of personality [38]), recognizing that personality is potentially a source of association between alcohol and sexual risk behaviour [39], and conscientiousness is strongly associated with both sexual risk behaviour [40] and binge drinking [41], due to a common association with self-control [42]. Each male is also assigned a gender-inequitable norm score, which is (loosely) the probability of endorsing inequitable gender norms (norms are not simulated in women as these are not consistently related to risk behaviour). More formally, the norm score is defined in terms of the Gender Equitable Men’s (GEM) scale [43], a scale based on attitudes to IPV, male sexual dominance, and gender roles in the home, which has been used in South Africa and elsewhere [15, 21, 44]. Due to lack of nationally representative GEM data, we use acceptance of IPV (wife beating), as measured in the 2016 Demographic and Health Survey (DHS) [45], as a proxy measure when determining the predictors of the inequitable gender norm score. A more detailed description of the modelling of personality and inequitable gender norms is provided in Sects. 1.1–1.2 of the supplementary materials.

We further assign to each individual aged 15 or older two alcohol consumption variables: the daily probability that they consume any alcohol, and the average number of drinks per day on which alcohol is consumed. The effects of demographic and socio-economic variables on these variables are estimated based on 2016 DHS data, and the effects of personality and inequitable gender norms are also included in the model. Self-reported alcohol consumption substantially understates true levels [46], and we therefore adjust the modelled consumption rates upwards to ensure consistency with South African alcohol sales data (for more detail see Sect. 1.3 of the supplementary materials). Individuals are classified as ‘binge drinkers’ if they consume at least 5 drinks per drinking day, at least once a month.

The model has also been extended to include casual once-off relationships (excluding contacts between sex workers and their clients). Although these casual relationships are often transactional in nature [8, 47], we do not model transactional sex explicitly. Individuals are assumed to progress through phases of casual sex activity, with rates of entry into these phases determined by their demographic, socio-economic and sexual behaviour characteristics (described more fully in Sect. 1.5 of the supplementary materials).

**Fig. 1:**
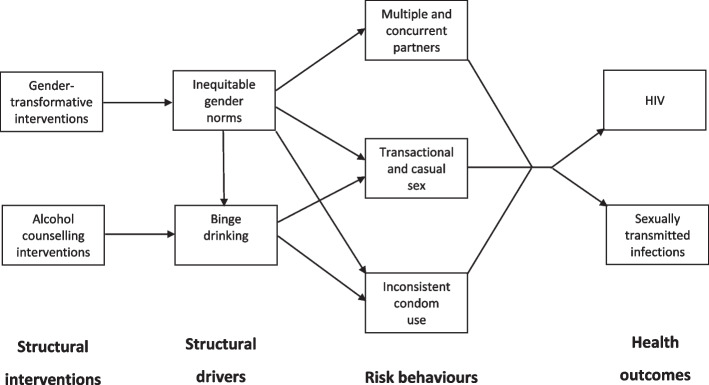
Conceptual model. Boxes represent model processes/variables, and arrows represent hypothesized causal relationships

Figure 1 summarizes the hypothesized effects of inequitable gender norms and binge drinking on sexual risk behaviour. The effect of binge drinking on HIV/STI risk is assumed to be mediated mainly by increased casual sex and reduced condom use, while the effect of inequitable gender norms (in men) is assumed to be mediated by increased partner concurrency and casual sex and negative attitudes to condom use, as well as increased binge drinking.

### Calibration and uncertainty analysis

Prior distributions are assigned to represent the uncertainty around the effects shown in Fig. 1 (summarized in Table 1). In most cases, hurdle distributions are specified, to allow a non-zero probability of a null effect, and the strength of evidence from observational studies and randomized controlled trials (RCTs) is reviewed in deciding the prior probability of a null effect. Prior distributions are also specified for parameters that represent potential sources of confounding. Specifically, we specify beta priors to represent the strength of association between risk group and binge drinking and between risk group and inequitable gender norms, independent of any causal relationship between the respective variables.

**Table 1:** Key model assumptions

Model parameters	Mean	SD	Data sources
Risk group assumptions			
% of women in ‘high risk’ group (propensity for concurrent partners)	25%	-	
% of men in ‘high risk’ group (propensity for concurrent partners)	35%	-	
Personality assumptions			
Decrease in odds of being high risk for each SD increase in conscientiousness score	0.67	-	[40, 79]
Decrease in drinks per drinking day, for each SD increase in conscientiousness score	0.32	-	[41]
Inequitable gender norm assumptions (men only)			
OR for association between ‘low risk’ group and inequitable gender norms, not modifiable by interventions	0.50	0.20	[80]
Effects of age, education, race, and urban/rural location on inequitable gender norms	Table S1	-	2016 DHS^a^
Effect of inequitable gender norms on incidence of concurrency	2.50	3.06	[15]
Relative rate of endorsing inequitable gender norms after			
Gender-transformative interventions at individual level	0.50	0.29	Vague prior
Gender-transformative interventions at community level	0.50	0.29	Vague prior
Annual probability of reverting to pre-intervention gender norms	0.50	0.29	Vague prior
Alcohol assumptions			
Effects of age, sex, education, employment, race, urban/rural, marriage on			
Daily probability of alcohol consumption	Table S2	-	2016 DHS^a^
Number of drinks per drinking day	Table S3	-	2016 DHS^a^
Increase in drinks per drinking day, comparing men who always endorse inequitable gender norms to those who never do	6.25	5.00	[15]
Ratio of reported drinking frequency (% days) to true drinking frequency	0.65	-	Calibrated to
Ratio of reported drinks per drinking day to true drinking volume	"	-	alcohol sales
Probability of confounding between risk group (propensity for concurrent partners) and number of drinks per drinking day	0.50	0.29	Vague prior
Relative rate of drinking (per day) after versus before intervention			
Single session of alcohol counselling	0.80	0.10	[23]
Multiple sessions of alcohol counselling	0.50	0.20	[23]
Annual probability of reverting to pre-intervention drinking levels	0.50	0.29	[81–83]
Casual sex assumptions			
Annual rate of entry into casual sex state: single high-risk males aged 17	0.08	-	[8]
Annual rate of entry into casual sex state: single high-risk females aged 17	0.15	-	[3]
Relative rate of entry into casual sex state: single low-risk males	0.30	-	[8]
Relative rate of entry into casual sex state: single low-risk females	0.15	-	[3]
Relative rate of entry into casual sex state: male binge drinkers	1.38	0.43	[2, 6, 8, 12]
Relative rate of entry into casual sex state: female binge drinkers	2.15	1.35	[3–5, 12]
Relative rate of entry into casual sex state per 0.1 decrease in inequitable gender norm score (men only)	0.88	0.19	[14, 17]
Condom use assumptions			
Reduction in odds of condom use, per day of binge drinking/week (OR)	0.83	0.23	[1, 7]
Relative rate of condom use, comparing men who always endorse inequitable gender norms to those who never do	0.63	0.39	[10, 19]

*OR* Odds ratio, *SD* Standard deviation.

^a^Own analysis (see supplementary materials)

The model is calibrated to RCT data, from trials of alcohol counselling and gender-transformative interventions conducted in sub-Saharan Africa. Trials were identified from recent systematic reviews [24, 48–50]. Alcohol counselling RCTs were included if they recruited individuals who were hazardous drinkers at baseline, and were classified as involving either a single or multiple alcohol counselling sessions. Gender-transformative interventions were included if the intervention involved critical reflection of gender norms and gender inequalities, and because we only modelled the effect of inequitable gender norms on male risk behaviours, trials were excluded if they only reported effects in women. We distinguished between gender-transformative interventions that aimed to change gender norms through individual or group counselling and those that aimed to change gender norms at a community level, typically through community mobilization [48]. Nine alcohol counselling interventions (5 single-session, 4 multiple-session) and 4 gender-transformative interventions (2 individual-level, 2 community-level) were included (Supplementary Table S7). For each of the four types of intervention, a single efficacy parameter was specified (for the binge drinking/inequitable norm outcome) and prior distributions were specified to represent the uncertainty around each efficacy parameter (Table 1). In addition, we specified parameters that determine the extent to which individuals revert to their baseline behaviours after initially changing their behaviours in response to the intervention. A more detailed description of the prior distributions and the data sources on which they are based is provided in Sect. 3.1 of the supplementary materials.

To calibrate the model, we drew an initial sample of 5000 parameter combinations from the prior distributions in Table 1. For each combination of parameters sampled, we ran the model ten times: twice to estimate outcomes in a ‘no intervention’ scenario and then twice for each of the four types of intervention, in each case assigning the intervention to all individuals eligible to receive it (the model was run twice with different random numbers in order to quantify the extent of stochastic variation in model outcomes). Intervention effects were calculated for each trial outcome (changes in alcohol consumption, gender norms, sexual risk behaviour, HIV incidence and STI incidence), at different intervention durations and in different sub-populations (defined by age and sex). For each parameter combination, a likelihood value was calculated to represent the consistency between the simulated trial outcomes and actual observed trial outcomes (see Sect. 3.2 of the supplementary materials). From the initial set of 5000 parameter combinations, we selected the 50 parameter combinations with the highest likelihood values and used these to generate more detailed model outputs (means and 95% confidence intervals). Sub-analyses specific to a particular intervention (or group of interventions) were performed by selecting the 50 parameter combinations that yielded the highest likelihood for the corresponding subset of the RCT data. The model was validated by comparing the modelled associations between binge drinking, inequitable gender norms and sexual risk behaviour, with the associations observed in four nationally representative household surveys in 2008, 2012, 2016 and 2017 [51, 52].

### Model analyses

In all analyses, HIV transmission probabilities and STI parameters were fixed at the average of the best-fitting values estimated when the model was previously calibrated to South African HIV prevalence data [37] and STI prevalence data [53]. For the purpose of reporting STI incidence outcomes we summed new cases of gonorrhoea, chlamydia and trichomoniasis, as these are the curable STIs that occur most frequently in South Africa. Population attributable fractions (PAFs, or proportions of incident HIV and STI infections attributable to binge drinking and inequitable gender norms) were estimated by comparing the HIV and STI incidence estimates in the base scenario with counterfactual scenarios in which (a) binge drinking was assumed to have no effect on sexual risk behaviour from 2000, and (b) all gender-inequitable norm scores were set to zero from 2000. Intervention impacts were estimated for scenarios in which interventions were introduced from mid-2021. In sensitivity analyses, intervention impacts were estimated under the optimistic assumption of no reversion to baseline behaviours, i.e. approximating what might be expected if the intervention were repeated at regular intervals rather than being once-off.

## Results

### Base scenario: HIV, STIs and risk behaviours

**Fig. 2:**
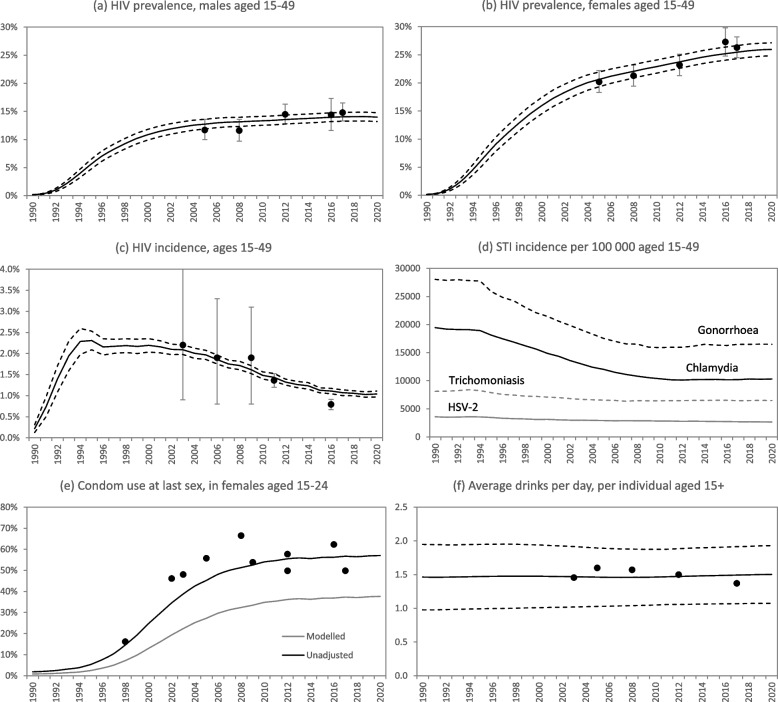
Model validation in the base scenario. In all panels (except panels d and e) the solid line represents the average outputs from 50 model simulations and the dashed lines represent the 95% confidence intervals. In panels a-c and e, dots represent results from national household surveys [51, 52, 84–90], with error bars representing 95% confidence intervals. In panel f, dots represent data from national surveys of alcohol use [52, 84, 85, 90–92]. In panel e, the ‘unadjusted’ estimate of condom use is calculated from the modelled rate of condom use, applying an odds ratio of 2.2, i.e. assuming the odds of self-reported condom use is 2.2 times the true odds of condom use (the model being lower than the survey data to account for social desirability bias)

The model is validated against HIV prevalence data and incidence estimates from national household surveys (Fig. 2a-c). HIV and STI incidence rates are estimated to have declined since the late 1990s (Fig. 2c-d), largely as a result of increases in condom use (Fig. 2e) and – in the case of HIV – increased uptake of HIV testing and ART [29]. Estimates of average daily alcohol consumption are high, and in line with alcohol sales data (Fig. 2f). The model estimates that binge drinking is highly prevalent in South Africa: the proportion of adults aged 15–49 who were classified as binge drinkers in 2021 was 54% in men and 35% in women. Inequitable gender norms are estimated to be particularly common in younger men: the average gender-inequitable norm score in 2021 was 0.34 in males aged 15–24, compared to 0.24 in males aged 25–34 and 0.16 in males aged 35 and older (the average score for all males aged 15 and older was 0.22).

### Calibration to RCT data

The distributions of best-fitting parameters are compared to the prior distributions in Table S8. Model estimates of intervention effectiveness, obtained using the 50 best-fitting parameter combinations, were generally within the 95% confidence intervals around the observed trial outcomes (Supplementary Figures S6, S7 and S8). The model also performed reasonably when validated against South African household survey data, matching the strong observed associations between inequitable gender norms, binge drinking and multiple partnerships, and matching the relatively weak observed associations between binge drinking and condom use (Supplementary Figure S9).

### Population attributable fractions

Over the period from mid-2000 to mid-2021, an estimated 54% (95% CI: 34–74%) of new HIV infections in adults occurred in binge drinkers (67% of new infections in men and 46% of new infections in women). However, the proportion of incident HIV infections over the same period that were attributable to binge drinking was relatively small (6.8%, 95% CI: 0.0–32.1%). Similar PAFs were estimated when men and women were considered separately, and when considering the proportion of incident STIs attributable to binge drinking (Table 2). The parameter most strongly affecting these PAFs was the effect of binge drinking on women engaging in casual sex (Table 3).

**Table 2:** Proportion of incident HIV and STI infections (2000–2020) attributable to binge drinking and inequitable gender norms

	HIV incidence			STI incidence		
	Total	Male	Female	Total	Male	Female
Binge drinking	6.8% (0.0–32.1)	7.3% (0.0–34.0)	6.4% (0.0–29.9)	7.6% (0.0–37.3)	7.8% (0.0–36.7)	7.3% (0.0–36.7)
Inequitable gender norms	17.5% (0.0–68.3)	15.8% (0.0–62.4)	19.1% (0.0–72.4)	17.3% (0.2–58.3)	16.8% (0.4–54.5)	17.4% (0.1–63.5)
Binge drinking and inequitable gender norms	24.5% (0.7–63.1)	22.9% (0.9–57.9)	25.6% (0.6–66.5)	22.4% (0.5–60.9)	21.9% (0.6–58.6)	23.0% (0.3–64.5)

**Table 3:** Correlation between model parameters and population attributable fractions

Parameter	HIV incidence			STI incidence		
	Total	Male	Female	Total	Male	Female
*Incidence attributable to binge drinking*						
OR of condom use, per day of binge drinking, per week	-0.16	-0.12	-0.19	-0.08	-0.09	-0.05
Increase in casual sex in						
Male binge drinkers	0.23	0.27	0.20	0.23	0.24	0.21
Female binge drinkers	**0.85**	**0.86**	**0.83**	**0.88**	**0.88**	**0.89**
*Incidence due to inequitable gender norms*						
Effect of inequitable gender norms on						
Men’s entry into concurrent partnerships	**0.85**	**0.80**	**0.87**	**0.82**	**0.79**	**0.86**
Men’s number of drinks per drinking day	0.08	0.07	0.10	0.05	0.02	0.09
RR condom use in men endorsing inequitable gender norms	**-0.41**	**-0.43**	**-0.40**	**-0.37**	**-0.38**	**-0.34**
RR of entry into casual sex per 0.1 decrease in gender inequitable norm score	0.03	-0.02	0.06	-0.10	-0.13	-0.05

*RR* Relative risk. Correlation coefficients in bold are statistically significant

Over the 2000–2021 period, an estimated 17.5% (95% CI: 0.0–68.3%) of new HIV infections in adults were attributable to inequitable gender norms. PAFs were similar when considering HIV incidence in men and women separately, and when considering incident STIs (Table 2). The most significant parameter determining the proportion of incident infections attributable to inequitable gender norms was the effect of inequitable gender norms on male partner concurrency, and the effect of inequitable gender norms on male condom use was also significant (Table 3). Overall, inequitable gender norms and binge drinking together accounted for 24.5% (95% CI: 0.7–63.1%) of incident HIV cases over the 2000–2021 period.

### Intervention effects

The model calibration to the RCT data suggests that multi-session alcohol counselling interventions are substantially more effective than single-session interventions in reducing alcohol consumption (Table S8). Individual-level gender-transformative interventions appear more effective than community-level interventions in reducing inequitable gender norms in the short term, but there is a significantly more rapid reversion to baseline gender norms in the former case.

**Fig. 3:**
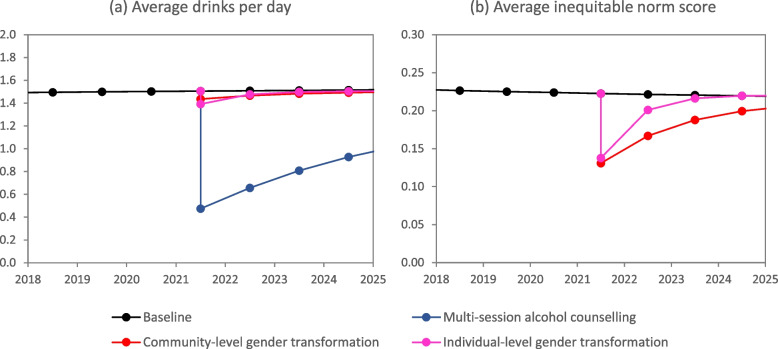
Impact of interventions on levels of alcohol consumption and probability of endorsing inequitable gender norms

A multi-session alcohol counselling intervention that reached all binge drinkers in South Africa, implemented in 2021, would be expected to reduce substantially the average number of drinks per day in the short term, but the impact would be less over the longer term, as those exposed to the intervention revert to their baseline drinking levels (Fig. 3a). Over the 5 years following the intervention, the total number of new HIV infections would be reduced by only 1.2% (95% CI: 0.0–2.5%), compared to the status quo scenario, and the impact on STI incidence would also be only a 1.3% reduction (95% CI: 0.0–3.7%). Intervention impacts would be similar in women and men (Table 4). However, the reduction in STI incidence would be marginally greater if the intervention impact did not wane over time (1.9%, 95% CI: 0.0–4.5%).

**Table 4:** Projected reductions in HIV and STI incidence under different intervention scenarios (2021–2026)

	HIV incidence			STI incidence		
	Total	Male	Female	Total	Males	Female
Multi-session alcohol counselling						
With waning impact	1.2% (0.0–2.5)	1.3% (0.0–2.5)	1.2% (0.0–2.4)	1.3% (0.0–3.7)	1.3% (0.0–3.4)	1.4 (0.0–3.7)
No waning	1.2% (0.0–2.2)	1.5% (0.0–3.9)	1.1% (0.0–1.7)	1.9% (0.0–4.5)	1.9% (0.0–4.5)	1.9% (0.0–8.9)
Community-level gender transformative intervention						
With waning impact	3.2% (0.8–7.2)	3.2% (0.1–10.7)	3.2% (0.3–9.4)	2.4% (1.1–4.2)	2.2% (1.0–3.8)	2.7% (1.5–4.4)
No waning	7.3% (0.6–21.2)	4.3% (0.1–14.9)	9.1% (0.9–25.1)	6.4% (1.2–15.2)	5.7% (1.2–13.1)	7.5% (1.1–19.4)
Individual-level gender ransformative intervention						
With waning impact	0.7% (0.4–1.2)	0.3% (0.0–1.1)	1.1% (0.2–2.8)	0.6% (0.3–1.2)	0.6% (0.1–1.5)	0.7% (0.2–1.7)
No waning	2.4% (0.2–6.9)	2.5% (0.4–6.6)	2.2% (0.2–6.5)	2.5% (0.3–6.7)	2.1% (0.3–5.5)	3.3% (0.2–10.3)

A gender-transformative intervention that reaches all men, implemented in 2021, would be expected to achieve large short-term reductions in men’s average gender-inequitable norm scores, regardless of whether the intervention is introduced at the individual or community level (Fig. 3b). It would also have a modest impact on levels of alcohol consumption (Fig. 3a). However, male endorsement of inequitable gender norms is predicted to revert to baseline more rapidly in the context of an individual-level intervention than would be expected in the context of a community mobilization intervention. As a result, a community-level gender-transformative intervention is expected to lead to a greater reduction in HIV incidence over 5 years (3.2%, 95% CI: 0.8–7.2%) than an individual-level gender-transformative intervention (0.7%, 95% CI: 0.4–1.2%). Similar impacts are predicted for STI incidence (Table 4). However, the expected reduction in HIV incidence is substantially greater if the effects of gender-transformative intervention do not wane (7.3% [95% CI: 0.6–21.2%] for community-based interventions and 2.4% [95% CI: 0.2–6.9%] for individual-level interventions).

## Discussion

Our results suggest that the nexus between inequitable gender norms, binge drinking and sexual risk behaviour accounts for around 25% of HIV incidence in South Africa over the last two decades. Binge drinking is estimated to be highly prevalent after adjusting for under-reporting of alcohol consumption in national household surveys. Although inequitable gender norms are less prevalent, they nevertheless exert a stronger influence on HIV and STI incidence. Despite the substantial proportion of incident HIV attributable to inequitable gender norms and binge drinking, recently-trialled interventions that aim to reduce these structural drivers through counselling and community mobilization are estimated to have only a modest impact on HIV incidence at a national level.

We have shown that more than half of incident HIV infections occur in binge drinkers, consistent with studies in other African settings, which have estimated the proportion of HIV incidence associated with alcohol to be as high as 64% [54, 55], and which have found as many as 87% of new sexual partners are met in drinking venues [56]. Previous reviews have noted the strong association between HIV and alcohol consumption in sub-Saharan Africa [57–59]. Although it is difficult to tease out how much of this association is causal, and although our findings suggest that a relatively modest fraction of incident infections in binge drinkers may be directly attributable to heavy alcohol use, it is nevertheless important to recognize heavy alcohol consumers as a ‘key population’, in which a high proportion of incident HIV and STI infection occurs. Regardless of whether structural interventions are able to substantially shift levels of binge drinking, it is important to also target more traditional HIV interventions to patrons of drinking establishments [56].

To the extent that there is a true effect of binge drinking on HIV incidence, this appears to be mediated largely by its effect on casual sex in women. Although binge drinking is more common in men than in women, it may be a greater risk factor for casual/transactional sex in women than in men [3, 5, 6, 12]. In our model, male demand for casual sex exceeds female demand for casual sex, and this might also explain why the modelled HIV incidence is more sensitive to changes in female demand for casual sex than to changes in male demand. Although binge drinking may have an effect on unprotected sex, our results suggest that the more significant effect (at least in women) is that regular binge drinking increases the likelihood of casual/transactional sex encounters, probably directly through drinking venues.

Our results suggest that the substantial impact of inequitable gender norms on HIV incidence is largely mediated by the effect of inequitable gender norms on men’s engaging in concurrent relationships. Previous network modelling studies have demonstrated the profound importance of concurrent partnerships in sustaining HIV and STI transmission [53, 60–62], but have not identified the structural underpinnings of concurrency in the populations in which it is highly prevalent. Interventions to reduce inequitable gender norms may play an important role in reducing concurrency. Our results suggest that community mobilization interventions may be relatively more effective in the longer term than individual-focused interventions. This is consistent with studies showing that concurrency is strongly influenced by social networks [18], i.e. men may be more likely to reduce concurrency if they perceive a change in the accepted norms of their peers. In sensitivity analysis, even more substantial reductions in HIV incidence were achieved if it was assumed that there was no reversion to baseline behaviours, suggesting that community mobilization efforts would need to be sustained and repeated in order for substantial changes in risk behaviour to be achieved.

A general limitation is that our estimates of PAFs and intervention impacts are imprecise. For example, the 95% confidence interval around the proportion of HIV incidence attributable to binge drinking is 0–32%. We believe this is an honest reflection of the uncertainty that exists when taking into account the possible sources of confounding in cross-sectional and observational data, which we have not used in calibrating our model. We have instead relied only on RCT data in model calibration, which are more likely to reflect the ‘true’ causal relationships between binge drinking, inequitable gender norms and sexual risk behaviour. However, even RCT data are imperfect, with most RCTs being under-powered to measure intervention effects on sexual risk behaviour (or HIV/STI incidence) with a high degree of precision, and this lack of precision is reflected in the wide confidence intervals around our estimates.

Another limitation of this study is that we have considered only the effects of binge drinking and inequitable gender norms on sexual risk behaviour. Alcohol and gender norms may affect HIV and STI dynamics through other mechanisms. For example, heavy alcohol consumption may compromise immunity, increasing susceptibility to HIV and STIs [63]. Binge drinking also negatively affects ART adherence [64], thus increasing HIV morbidity and mortality, as well as the risk of HIV transmission. Inequitable gender norms are also associated with poor health seeking in men [65, 66], and although evidence in women is inconsistent, some studies suggest gender-transformative interventions could reduce sexual risk behaviour in women [67, 68]. This means that the true proportion of HIV incidence and mortality attributable to binge drinking and inequitable gender norms may be greater than we have estimated.

We have also not considered here the effect of inequitable gender norms and binge drinking on IPV, which is an important health outcome in its own right. Gender-transformative interventions and alcohol counselling interventions can significantly reduce IPV [25, 69, 70]. Our previous modelling suggests that although there are strong associations between IPV and HIV, most observed associations between IPV and HIV in women can be attributed to confounding factors, and that interventions to reduce IPV alone are unlikely to have much impact on HIV incidence [71]. It has been argued that in order to reduce HIV incidence, interventions need to tackle inequitable gender norms and hegemonic masculinity more broadly, and that it is these inequitable gender norms that account for much of the confounding between IPV and HIV [71, 72].

Implications for alcohol policy are unclear. It is debatable whether it is feasible to conduct individual-level counselling interventions at a large scale, and whether such interventions would reduce binge drinking over the long term [73]. Our simulations of the impact of reaching the whole South African population with such interventions in a short space of time are not intended to be realistic, but illustrate what might be achieved in a ‘best case scenario’. Given that the simulated intervention impacts on HIV and STIs are modest even under these extremely optimistic assumptions, we would caution against the uncritical adoption of these recently-trialled interventions. Addictive behaviours are difficult to change, and many of the RCTs used in calibrating our model adopted a ‘harm reduction’ approach, aiming for reductions in alcohol use to safe levels and reductions in associated sexual risk, rather than complete abstinence. Interventions that promote self-help groups, which typically aim for complete abstinence, may also be effective [74], and would probably be less expensive. Other structural interventions, such as minimum unit pricing [75], increased alcohol taxation [76], improved enforcement of existing legislation around alcohol sales [76] and school-based interventions [77], may also prove more cost-effective and feasible in a resource-limited setting, and should be explored.

Further research is required to measure inequitable gender norms at a national level, in South Africa and elsewhere. DHS questionnaires only include questions about endorsement of IPV, and do not assess other components of inequitable gender norms. Given the importance of inequitable gender norms in driving HIV and IPV, and given the need to monitor the impact of gender-transformative interventions at a national level, it is critical that assessments of inequitable gender norms are included in future national household surveys. Beliefs about gender norms are most frequently formed during childhood and adolescence, with parents, school environments and religious instruction all playing key roles [78]; gender transformative interventions should therefore engage more actively with these sources of influence. Female economic empowerment may also shift gender norms, particularly in relation to IPV [78], and effects on inequitable gender norms need more thorough evaluation. Interventions that address gender norms around alcohol consumption may also be particularly important in reducing hazardous drinking.

## Supplementary Information

**Additional file 1: Supplementary materials. Table S1. **Multivariable analysis of factors associated with men’s acceptance of wife beating. **Figure S1.** Modelled trend in average gender-inequitable norm scores. **Table S2.** Predictors of the numbers of days on which alcohol was consumed in the last week (on a log scale). **Table S3.** Predictors of the average number of drinks per drinking day, among adults who consumed alcohol in the last week (on a log scale). **Table S4.** Variation in numbers of days on which alcohol is consumed and numbers of drinks per drinking day. **Figure S2.** Average drinks per day, for individuals aged 15 and older. **Figure S3.** Proportion of adults aged 15 and older who consume alcohol at least monthly. **Figure S4.** Proportion of high school students (grades 8-11) who have had 5 or more drinks on a single day in the last month. **Table S5.1.** Model assumptions about heterosexual rates of entry into the casual sex state. **Table S5.2**. Heterosexual casual sex model outputs in 2005.** Figure S5.** Changes in the relationship between educational attainment and condom use over time. **Table S6.** Prior distributions for alcohol and gender norm parameters. **Table S7.** Randomized controlled trial data included in model calibration. **Table S8.** Best-fitting parameter combinations. **Figure S6.** Calibration to alcohol and gender norm outcome data. **Figure S7**. Calibration to sexual risk behaviour outcome data. **Figure S8.** Calibration to biological outcome data. **Figure S9.** Modelled associations (on log(OR) scale) between binge drinking, inequitable gender norms and sexual risk behaviours, compared against associations measured in national surveys. **Table S9.** Changes in condom parameters. **Figure S10.** HIV prevalence in adults aged 15-49. **Figure S11.**  Annual AIDS deaths. 

## Data Availability

The randomized controlled trial data that are used in model calibration are available as part of the supplementary materials (PublicDataRCTs.xlsx). The 2016 South African DHS data are freely available from https://dhsprogram.com/methodology/survey/survey-display-390.cfm. The C +  + code used is available from the corresponding author on request.

## Abbreviations

ABM: Agent-based model
ART: Antiretroviral treatment
DHS: Demographic and Health Survey
GEM: Gender Equitable Men’s (scale)
HIV: Human immunodeficiency virus
IPV: Intimate partner violence
MicroCOSM: Microsimulation for the Control of South African Morbidity and Mortality
PAF: Population attributable fraction
RCT: Randomized controlled trial
SAVA: Substance abuse, violence and AIDS
STI: Sexually transmitted infection
