# Supplementary material for: An agent-based model of binge drinking, inequitable gender norms and their contribution to HIV transmission, with application to South Africa
Source: BMC Infect Dis. 2023 Jul 29;23:500. doi: 10.1186/s12879-023-08470-y (PMC10385913; doi:10.1186/s12879-023-08470-y)
Supplement: Supplementary file 1 — Additional file 1: Supplementary materials. Table S1. Multivariable analysis of factors associated with men’s acceptance of wife beating. Figure S1. Modelled trend in average gender-inequitable norm scores. Table S2. Predictors of the numbers of days on which alcohol was consumed in the last week (on a log scale). Table S3. Predictors of the average number of drinks per drinking day, among adults who consumed alcohol in the last week (on a log scale). Table S4. Variation in numbers of days on which alcohol is consumed and numbers of drinks per drinking day. Figure S2. Average drinks per day, for individuals aged 15 and older. Figure S3. Proportion of adults aged 15 and older who consume alcohol at least monthly. Figure S4. Proportion of high school students (grades 8-11) who have had 5 or more drinks on a single day in the last month. Table S5.1. Model assumptions about heterosexual rates of entry into the casual sex state. Table S5.2. Heterosexual casual sex model outputs in 2005. Figure S5. Changes in the relationship between educational attainment and condom use over time. Table S6. Prior distributions for alcohol and gender norm parameters. Table S7. Randomized controlled trial data included in model calibration. Table S8. Best-fitting parameter combinations. Figure S6. Calibration to alcohol and gender norm outcome data. Figure S7. Calibration to sexual risk behaviour outcome data. Figure S8. Calibration to biological outcome data. Figure S9. Modelled associations (on log(OR) scale) between binge drinking, inequitable gender norms and sexual risk behaviours, compared against associations measured in national surveys. Table S9. Changes in condom parameters. Figure S10. HIV prevalence in adults aged 15-49. Figure S11. Annual AIDS deaths. [file 12879_2023_8470_MOESM1_ESM.pdf]

Supplementary materials

Contents

1. Extensions to the MicroCOSM model structure 2
1.1 Modelling personality 2
1.2 Modelling inequitable gender norms 4
1.3 Modelling alcohol use 7
1.4 Modelling concurrent partnerships 15
1.5 Modelling casual sex 16
1.6 Modelling condom use 20
1.7 Modelling alcohol counselling interventions 23
1.8 Modelling gender transformative interventions 25
2. Method for estimating confidence intervals 26
2.1 Additional complications when there is a non-zero probability that $p = 0$ 27
3. Calibration to randomized controlled trial data 27
3.1 Prior distributions 28
3.2 Likelihood function 29
3.3 Selecting the best-fitting parameter combinations 34
3.4 Best-fitting parameter combinations 34
3.5 Calibration outputs 36
4. Additional results 39
4.1 Validation against household survey data 39
4.2 Calibration to HIV data 41
References 45

1. Extensions to the MicroCOSM model structure

The structure of the MicroCOSM model and its assumptions has been described in detail previously [1]. The sections that follow describe recent extensions to the model structure that are relevant to modelling the role of alcohol and inequitable gender norms in the epidemiology of HIV in South Africa.

1.1 Modelling personality

Personality has been shown to be a significant determinant of engagement in health risk behaviours and in health seeking, as well as being important in determining socio-economic status. It is therefore important to understand and model the role of personality if one is to control for possible confounding when evaluating associations between diseases and health risk behaviours.

Several models have been developed to describe personality traits. The most widely used is the Five Factor Model, which characterizes individuals according to five broad sets of characteristics: conscientiousness, extraversion, openness to experience, agreeableness, and 'neuroticism' (emotional instability) [2]. Although all five of these factors influence health risk behaviour, the factor that consistently stands out as being most strongly associated with health risk behaviours and health outcomes is conscientiousness [3, 4]. Studies have shown that low levels of conscientiousness are strongly associated with alcohol consumption [5, 6], sexual risk behaviour [7, 8] and lower socioeconomic status [9, 10].

In modelling conscientiousness, we assign to each individual in our simulated population a conscientiousness score. Although there have been several studies measuring dimensions of personality in South Africa [11-13], none of these studies describe a nationally representative sample of the South African population, with most being conducted in university students or employed individuals (i.e. individuals of relatively high socio-economic status). We therefore do not base the assignment of the conscientiousness score on any local data; rather the conscientiousness score represents the difference in conscientiousness, relative to the national average, expressed in terms of standard deviations. Thus a conscientiousness score of zero means the individual has a conscientiousness the same as the national average, while an individual with a conscientiousness score of 2 is 2 standard deviations above the national average in terms of their level of conscientiousness (assuming that scores are normally distributed, this means they are approximately at the 98th percentile of conscientiousness scores in the population).

Levels of conscientiousness appear to be relatively similar across populations [14]. Evidence regarding the role of age in determining conscientiousness is inconsistent. Costa and McCrae [15] argue that personality tends to remain stable over the adult life course, and in a large US study, age was not found to be significantly associated with conscientiousness [3]. On the other hand, in a meta-analysis of longitudinal studies, Roberts and Walton [16] estimated that conscientiousness tends to increase over the life course, by an average of 0.18 standard deviations per 10-year increase in age. They argued, however, that although average levels of conscientiousness change over the life course, *relative* differences between individuals tend to be consistent over time (i.e. when compared to other individuals of the same age, a person's personality tends to be stable over time). They also acknowledged that there was a lack of data on personality development in African and Asian countries. In the interests of simplicity, and

given the lack of locally-relevant data, we do not model age-related changes in individuals' conscientiousness score, though we note that this may limit our model's ability to explain certain age-related changes in risk behaviour.

Levels of conscientiousness appear inversely related to levels of sexual risk behaviour in the international literature. Schmitt [7] found, in a global study of associations between personality and sexual behaviour, that higher conscientiousness was negatively associated with relationship infidelity, both in men (r = -0.17) and women (r = -0.20), and with associations observed in the African region being similar (r = -0.20 in men and -0.12 in women). In a recent meta-analysis, Allen and Walter [8] estimated a similar negative association between conscientiousness and sexual infidelity (r = -0.17). In our model we define individuals as 'high risk' if they have a propensity for concurrent partnerships or commercial sex activity. We assume that for individuals who have a zero conscientiousness score (i.e. average conscientiousness), the probability of being in the high-risk group is 0.35 for males and 0.25 for females. We further assume that for each unit increase in conscientiousness, the odds of being in the high-risk group decreases by a factor of 0.67. This means, for example, that the odds of being 'high-risk', for an individual who is at the 97.5 percentile of the conscientiousness scale, relative to that in someone at the 2.5 percentile of the conscientiousness scale, is 0.21 ($0.67^{(1.96 \times 2)}$). With these assumptions, the simulated correlation between the conscientiousness score and the individual's high-risk status is -0.19 in men and -0.17 in women (i.e. roughly consistent with the correlation coefficients estimated in the literature).

Conscientiousness is also assumed to affect levels of binge drinking, as explained in section 1.2 of the supplementary materials. Binge drinking is in turn assumed to influence a number of sexual risk behaviours, as outlined in the sections that follow.

Levels of conscientiousness correlate strongly with educational achievement. In a meta-analysis of studies that examined the association between traits in the Five Factor Model and academic performance, Poropat [9] found that openness and conscientiousness were the two dimensions of personality that correlated most strongly with academic performance (with r = 0.19 in the case of conscientiousness). In most of these studies, academic performance was quantified in terms of test scores or GPA, but in our model we are interested in calculating the effect of conscientiousness on the probability of grade repetition (which is a function of test scores). In a simulation of test scores, we found that if we assumed test scores increase on average by 0.20 standard deviations for each unit increase in conscientiousness score, the simulated correlation between conscientiousness and test score was 0.20 (i.e. very similar to the correlation found in the meta-analysis of Poropat [9]). In this simulation, the relative risk of grade repetition (i.e. a test score below the threshold for passing) reduced by factors 0.61-0.76 per unit increase in conscientiousness, depending on the level at which the threshold for passing was set. We therefore assume that the probability of grade repetition reduces by a factor of 0.70 per standard deviation increase in conscientiousness. We further assume that the probability of enrolment in tertiary education increases by a factor of 1.10 per unit increase in conscientiousness (based on the rates from the same simulation model if we set the threshold for advancement to tertiary education at the 70th percentile of test scores). However, we assume conscientiousness has no effect on rates of completing tertiary education. This is consistent with the meta-analysis of Poropat [9], which found that most measures of personality ceased to be significant predictors of academic performance at tertiary level, a finding that the author attributes to 'range restriction' (i.e. students who enrol in tertiary education are a 'select' group whose personality profiles are not typical of those in the general population, and we would

therefore not expect to see personality influencing their academic performance to the same extent).

Our model of the association between educational attainment and conscientiousness has some limitations. Firstly, it relies on an international meta-analysis, which is heavily weighted towards data from high-income countries, and such data may be less applicable in the South African setting. It has been noted, for example, that in poorer South African schools, rates of grade advancement are only weakly correlated with literacy and numeracy scores, with grade advancement in these settings being characterized as "a lottery" [17]. Under such circumstances, conscientiousness might not be strongly associated with grade advancement. Another limitation is that we have not attempted to model the relationship between conscientiousness and school dropout or university dropout. There is limited research on the relationship between school dropout and conscientiousness, with one US study finding that although conscientiousness was significantly negatively associated with school dropout, this association ceased to be significant after controlling for other personality traits (emotional stability and extraversion) [18].

1.2 Modelling inequitable gender norms

Various scores have been proposed to measure gender inequitable norms. The Gender Equitable Men's (GEM) scale is a scale based on attitudes to intimate partner violence (IPV), male sexual dominance, and gender roles in the home, which has been used in South Africa and elsewhere [19-21]. The Inequitable Gender Norms (IGN) scale, which is a sub-scale of the GEM scale, has also been evaluated in Ghana and Tanzania, and the different components of the scale have been shown to have high internal validity [22]. Shannon *et al* [23] consider a scale similar to the GEM scale, which measures attitudes to IPV, male partner concurrency and sexual control, and male education over female education.

Although we have some information on gender inequitable norms in samples of men in Mpumalanga [19] and KwaZulu-Natal [24], we lack nationally representative data on the prevalence of gender inequitable norms in South Africa and the factors that are associated with these norms. The nearest we are able to come to nationally representative data is the 2016 DHS, which included questions about attitudes towards IPV [25]. (Other components of the GEM scale were not evaluated directly, and questions about household decision making were asked only in married/cohabiting individuals.) In this survey, questions were asked about different circumstances in which a man might be justified in beating his wife. 9.2% of men aged 15-49 responded that it would be acceptable for a man to beat his wife under at least one circumstance, with the prevalence being highest in younger men, unmarried men, African men, men who had not completed secondary school and men living in rural areas (results of the multivariable analysis are summarized in Table S1, and are similar to those obtained in a previous analysis of the same data [25]). These risk factors are roughly consistent with international literature, which also tends to show a strong association between men's inequitable gender norms and younger age, being unmarried, being less educated and rural location [20, 26]. It is also consistent with a 2008-9 survey in Eastern Cape and KwaZulu-Natal, which found that the GEM scale was strongly associated with education, income and race [20]; and with a 1998 survey amongst municipal workers in Cape Town, which found that male reporting of violence against partners was significantly associated with age, education and race [27].

Table S1: Multivariable analysis of factors associated with men's acceptance of wife beating

Variable	Adjusted OR	95% confidence interval
Age group		
15-24	1	
25-34	0.63	0.46-1.28
35-44	0.53	0.38-0.74
45+	0.39	0.27-0.57
Education		
Primary	1	
Incomplete secondary	0.90	0.68-1.20
Completed secondary	0.51	0.36-0.74
Location		
Urban	1	
Rural	1.68	1.32-2.13
Race		
African	1	
Coloured	0.77	0.46-1.28
White/Asian	0.29	0.09-0.94

Source: 2016 DHS (author's own calculations)

In our model, we assign to each man a gender-inequitable norm score between 0 and 1, which can be crudely thought of as the individual's probability of endorsing or enacting gender-inequitable norms in a particular situation (note that this is negatively related to the GEM scale, which represents how *equitable* the individual's attitudes and beliefs are). At the start of the simulation, and for males reaching age 10, we use the above logistic regression model to randomly assign an initial gender-inequitable norm score. If R_i is a value randomly sampled from the uniform (0, 1) distribution for individual i, then the gender-inequitable norm score assigned to individual i is

$$G_i = \frac{1}{1 + exp\big(R_i - \beta x_i - \gamma(j_i - 1.65)\big)}$$

where x_i is the covariate vector for individual i, β represents the coefficients in Table S1 (on a log scale), j_i represents the risk group of individual i (1 if high-risk, 2 if low-risk) and exp(γ) represents the odds of gender-inequitable norms in low-risk men relative to high-risk men. (Since 35% of men are assumed to be high-risk, 1.65 represents the 'average' risk group.) We have assigned to exp(γ) a beta prior with a mean of 0.5 and a standard deviation of 0.2. (This is based on a Cape Town study, which found a 3-fold increase in the odds of IPV perpetration in men who had concurrent partners [27], and consistent with a literature review of similar studies in other settings [28].) Thus if the randomly sampled value R_i is 0.5 and exp(γ) is also 0.5, the gender-inequitable norm score assigned to an individual in the baseline category (urban African males aged 15-24 with no secondary education) would be 0.49 ($1/(1 + \exp(0.5)\times 0.5^{0.65})$) if high-risk and 0.32 ($1/(1 + \exp(0.5)\times 0.5^{-0.35})$) if low-risk. Based on the above regression model, it is assumed that for each 10-year increase in age, the odds of endorsing gender-inequitable norms reduce by a factor of 0.73.

It is worth noting that the exp(γ) term is included to represent the *association* between inequitable gender norms and concurrency, that is not modifiable by gender-transformative interventions, i.e. changes in inequitable gender norms would not lead to changes in this long-term risk group assignment. We make a separate provision for the direct effect of inequitable

gender norms on the rate of concurrency, through the θ parameter described in section 1.4. Because it is difficult to separate out how much of the observed association between inequitable gender norms and concurrency is due to a causal relationship, we assign prior distributions to represent the uncertainty in both the exp(γ) and θ parameters. The observed OR of 3, noted previously, might be considered an upper bound on exp(-γ), if all of the observed association was attributable to confounding factors and if gender-transformative interventions had no effect on concurrency. The prior mean of 0.5 corresponds to an OR of 2 for the degree of confounding in the observed association between gender-transformative norms and concurrency.

Similarly, it is important to note that the assumption that the norm score depends on age, education, location and race is not intended to represent an *effect* of these variables on gender norms but rather it is intended to capture the *associations* that are typically observed between socio-economic/cultural factors and gender-inequitable norms, which are often a reflection of the individual's social network rather than the characteristics of the individual himself. In future versions of the model, it may be helpful to move towards modelling these social network effects more explicitly, for example, modelling the effect at a household level of being exposed to IPV during childhood [27].

It is also worth noting that we do not account for the possibility that there might be variation between men in their gender-inequitable norm scores that is not attributable to the above-mentioned variables (age, education, race, urban/rural location and propensity for concurrency). This is because the regression model in Table S1 gives a coefficient of variation of 0.54 in predicted probabilities of endorsing inequitable gender norms. This compares with coefficients of variation in gender-inequitable norm scores of 0.44 locally [29] and 0.39-0.41 in other African settings [22]. Although these studies have been conducted in demographically homogeneous groups, and one might therefore expect less variation in gender norms than in a nationally representative survey, it would nevertheless appear that our model already accounts for a lot of inter-individual variation in gender norms (relative to that measured empirically), and further allowance for additional inter-individual variation is therefore probably not necessary.

Figure S1 shows the model estimates of changes over time in the average gender-inequitable norm score. As expected, the scores are negatively related with age. Within each age group, average scores are estimated to decline over time, which is due to both increasing urbanization and rising levels of education. The average inequitable norm score, for all males aged 15 and older, is around 0.2 in recent years. Although we do not have nationally representative data to validate these estimates, these results are roughly in line with studies in different South African settings. For example, the average inequitable norm score of around 0.3 in young men (ages 15-24) is midway between values measured in young men in Durban [30] and rural Mpumalanga [19], of 0.13 and 0.46 respectively (in both cases the reported inequitable norm scores were expressed as a proportion of the difference between the maximum and minimum possible scores.) Another study in older men in KwaZulu-Natal found a relatively high average inequitable norm score (0.42), but this could be because the study considered only HIV-positive adults in a mostly rural area [24], and rural location and HIV-positive status are both associated with more inequitable gender norms.

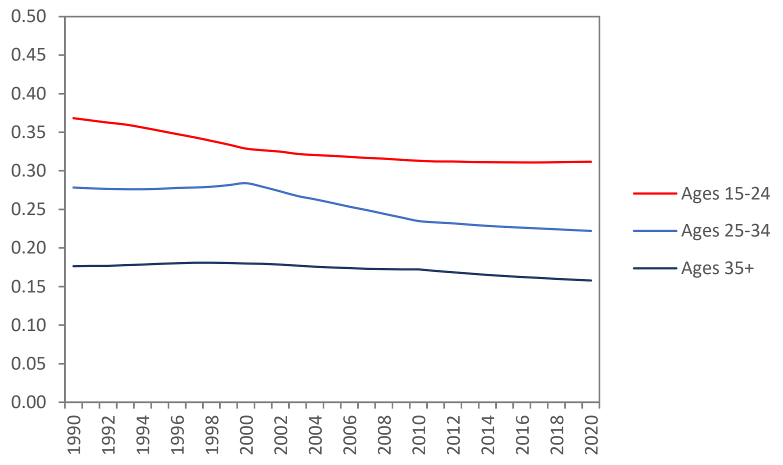

Figure S1: Modelled trend in average gender-inequitable norm scores

Gender-inequitable norm scores are not assigned to women. Although some studies suggest that women who accept inequitable gender norms are more likely to engage in concurrent partnerships [31], most RCTs of gender-transformative interventions have not been shown to reduce female reporting of multiple partners [30, 32] – probably because these interventions tend to have more impact on gender norms in men than in women [29, 30].

1.3 Modelling alcohol use

In our model, we assign to each individual two variables to represent their level of alcohol intake: the proportion of days on which they consume alcohol and the average number of drinks consumed per drinking day. The Alcohol Use Disorders Identification Test (AUDIT) assumes that one drink contains on average 10g of alcohol [33], although in South Africa an average drink more commonly contains 12g of alcohol [34]. With the modelled frequency of drinking and numbers of drinks per drinking day, it is possible to calculate the AUDIT-C score, which is based on the first three questions about alcohol intake in AUDIT [35]. For example, an individual who drinks on 10% of days and consumes an average of 5 drinks per drinking day would be categorized as having an AUDIT-C score of 6 (2 for their frequency of drinking [2-4 times a month] + 2 for their number of drinks per drinking day [5 or 6] + 2 for their frequency of binge drinking [monthly]). Because the remaining questions in the AUDIT questionnaire all relate to longer-term behaviours and experiences (over the last 12 months or longer), one would expect the short-term change in an individual's AUDIT score to be the same as the short-term change in their AUDIT-C score. This means that model assumptions about changes in the two alcohol intake variables following alcohol interventions can be validated against the short-term changes in AUDIT or AUDIT-C score that are reported in many randomized controlled trials of alcohol interventions [36-38].

Self-reporting of alcohol use is subject to social desirability bias, with individuals tending to under-report the extent of their alcohol intake. In South African surveys, the reported amount of alcohol consumed is typically only 12-22% of total national alcohol consumed [34, 39]. The proportion of South African youth who report recent binge drinking was found to be substantially higher in a 2008 survey that relied on self-administered questionnaires (33.5% in males, 23.7% in females) [40] than in a 2008 survey that relied on face-to-face interviews (7.6% in males, 2.6% in females) [41], suggesting that face-to-face interviews are particularly susceptible to social desirability bias. Phosphatidylethanol (PEth), a commonly used biomarker

of alcohol consumption over the last 3 weeks, is often detected in individuals who report no alcohol use. For example, Papas *et al* [42] found in Kenya that three months after enrolment 46% of subjects reported any alcohol intake over the last month, but this increased to 70% when including individuals who were PEth-positive. Similarly, Bajunirwe *et al* [43] found in Uganda that 21% of subjects reported drinking in the last 30 days, but this increased to 40% when including PEth-positive results. (These two studies imply that the odds of any alcohol consumption in the past 30 days is around 2.5-2.7 times the odds of reported alcohol use.) Studies have found that among PEth-positive individuals, those with higher PEth levels are more likely to report recent drinking [43, 44], and unmarried individuals were found to be more likely to report drinking in one study [44], but most other demographic and socio-economic variables do not appear to influence the extent of under-reporting [43, 44]. Some studies suggest greater under-reporting in women than in men. For example, a Tanzanian study found that PEth levels were more strongly correlated with self-reported alcohol consumption levels in men than in women [45] and a Ugandan study found that after controlling for levels of PEth, men reported higher levels of alcohol than women (though the difference was not statistically significant) [44]. The previously-noted discrepancies between self-reporting of alcohol use in South African youth, when comparing face-to-face interviews and self-completed questionnaires, are also suggestive of greater reporting biases in females than in males [40, 41].

Survey estimates of alcohol consumption may also be biased due to non-response bias. Individuals who were difficult to contact were more likely to report heavy drinking than individuals who were recruited after only a few contact attempts in a national UK survey, and similar results have been obtained in other settings [46]. The UK study found that non-response bias affected mainly the quantity of alcohol consumed per drinking day, not the daily probability of alcohol consumption. Studies suggest that questions about alcohol use in the last day (or alcohol on specific days in the last week) may yield more reliable reporting than questions about average alcohol use [47].

Setting assumptions about the extent of under-reporting is difficult because most studies focus only on under-reporting of any alcohol use (a binary outcome), whereas we wish to estimate the bias in terms of two continuous variables (frequency of drinking and number of drinks per drinking day). In the interests of simplicity, we assume that people under-report both variables by 17% in the DHS (our primary data source), in order to bring the model estimates in line with alcohol sales data (see below). This implies that the average ratio of reported alcohol intake to actual alcohol intake is 0.69 ((1 – 0.17) × (1 – 0.17)). This is higher than the coverage estimates of 12-22% noted previously, which is because we parameterize our model using 2016 DHS data on the number of units of alcohol consumed on each of the last 7 days. As noted previously, this approach to asking about alcohol use on specific days in the last week is thought to be less susceptible to bias than the more conventional approach of asking about average alcohol consumption [47].

1.3.1 Effect of socio-demographic factors on alcohol use

Table S2 shows the effect of various factors on the number of days on which alcohol was consumed in the last week, as assessed by applying a multivariable Poisson regression model to the data from the 2016 South African DHS data. Consistent with previous analyses of NIDS data [39], the reporting of alcohol consumption in men is greater at older ages, in unmarried men, in minority racial groups, and in men with higher educational attainment. Also consistent with the NIDS data, the reporting of alcohol consumption in women is most frequent at ages 25-34, in urban areas, and in minority racial groups. However, the NIDS data suggest a non-

significant relationship between urban location and alcohol consumption in men (in contrast to our finding of a weakly negative relationship), a significant positive relationship between being single and consuming alcohol in women (in contrast to our finding of a non-significant relationship) and a non-significant relationship between educational attainment and alcohol consumption in women (whereas our findings suggest women with tertiary education are more likely to report alcohol consumption). Employment was not a significant predictor of alcohol consumption, either in men or women (results not shown).

Table S2: Predictors of the numbers of days on which alcohol was consumed in the last week (on a log scale)

	Males		Females	
	Coefficient	95% CI	Coefficient	95% CI
Urban location	-0.08	-0.16 to -0.01	0.58	0.44 to 0.72
Age group (ref. 15-24)				
25-34	0.78	0.67 to 0.89	0.44	0.26 to 0.62
35-44	0.84	0.71 to 0.96	0.21	0.01 to 0.41
45-59	0.99	0.86 to 1.11	0.36	0.18 to 0.55
60+	0.98	0.84 to 1.12	0.27	0.07 to 0.48
Married/cohabiting	-0.33	-0.41 to -0.25	-	-
Education (ref. <secondary)				
Incomplete secondary	0.23	0.13 to 0.33	-0.05	-0.21 to 0.11
Completed secondary	0.36	0.24 to 0.47	0.03	-0.16 to 0.21
Tertiary	0.41	0.28 to 0.55	0.31	0.11 to 0.51
Race (ref. black African)				
Coloured	0.17	0.05 to 0.29	0.75	0.60 to 0.90
White or Asian	0.35	0.21 to 0.48	1.23	1.06 to 1.39
Constant	-1.03	-1.15 to -0.91	-2.48	-2.69 to -2.27

Source: 2016 DHS (author's own calculations)

Table S3 shows the predictors of the average number of drinks per drinking day, among those individuals who reported any alcohol consumption in the previous week. As before, this is based on applying a multivariable Poisson regression model to the data from the 2016 South African DHS. Consistent with a previous analysis of NIDS data [39], the amount of alcohol consumed per drinking day, among men who report drinking, is significantly lower among whites and Asians, thus offsetting their higher probability of reporting alcohol consumption on a given day (Table S2). Our results suggest similar racial differences in women, as well as a strong negative relationship between women's age and their average drinks per drinking day, again consistent with the NIDS data. Although our data suggest a similar negative relationship between age and average drinks per drinking day in men, the analysis of the NIDS data found the relationship not to be significant. Our results also suggest that the average number of drinks per drinking day is highest in urban areas for men, but in the NIDS analysis the effect of urban location was not significant. We also found the number of drinks per drinking day was greatest in men with secondary education, but in women completion of secondary education and tertiary education were weakly associated with *lower* numbers of drinks per drinking day; neither association was significant in the NIDS analysis. Marital status was not a significant determinant of the quantity of alcohol consumed per drinking day, either in men or women (results not shown).

Table S3: Predictors of the average number of drinks per drinking day, among adults who consumed alcohol in the last week (on a log scale)

	Males		Females	
	Coefficient	95% CI	Coefficient	95% CI
Urban location	0.08	0.04 to 0.13	-	-
Age group (ref. 15-24)				
25-34	0.10	0.04 to 0.16	-0.12	-0.22 to -0.01
35-44	-0.05	-0.11 to 0.02	-0.20	-0.33 to -0.07
45-59	-0.24	-0.31 to -0.17	-0.46	-0.60 to -0.32
60+	-0.42	-0.51 to -0.33	-0.85	-1.04 to -0.65
Education (ref. <secondary)				
Incomplete secondary	0.19	0.13 to 0.25	0.08	-0.05 to 0.22
Completed secondary	0.18	0.11 to 0.25	-0.15	-0.30 to 0.00
Tertiary	0.09	0.01 to 0.18	-0.17	-0.35 to 0.01
Employed	0.04	0.00 to 0.09	-	-
Race (ref. black African)				
Coloured	-0.03	-0.10 to 0.05	-0.06	-0.17 to 0.04
White or Asian	-0.62	-0.74 to -0.5	-0.57	-0.78 to -0.36
Constant	1.77	1.70 to 1.84	1.71	1.57 to 1.85

Source: 2016 DHS (author's own calculations)

Although other analyses have assessed correlates of alcohol consumption based on analyses of nationally representative survey data [41, 48], these are difficult to compare with our analysis because they do not separate out frequency of drinking episodes and amount of alcohol consumed per drinking episode. Nevertheless, our results are consistent with the finding in most South African studies that problem drinking is negatively related to educational attainment in women [41, 48-50] but unrelated to employment status in women [41, 49-51]. In men, findings on the relationship between socio-economic status and problem drinking have been less consistent across studies; our results suggest that there is a positive gradient at lower levels of education, but that tertiary education might be associated with a slightly reduced level of problem drinking.

1.3.2 Effect of personality on alcohol use

In an individual patient data meta-analysis of eight studies, Hakulinen *et al* [5] found that for each standard deviation increase in conscientiousness score, the odds of heavy drinking reduced by a factor of 0.89 (95% CI: 0.83-0.96), and the odds of transitioning from moderate to heavy drinking reduced by the same factor. However, conscientiousness had no significant effect on the odds of abstinence. This suggests that conscientiousness affects mainly the 'drinks per drinking day' variable in our model, rather than the proportion of days on which alcohol is consumed. Other personality traits, such as extraversion and neuroticism, were also strongly associated with heavy drinking, consistent with the findings of another review [6]. However, both reviews rely heavily on data from high-income settings, and it is not clear if personality factors associated with alcohol intake and binge drinking might be different in a South African setting.

1.3.3 Modelling alcohol use patterns

The regression model in Table S2 is used to assign to each individual aged 15 and older an *expected* daily probability of alcohol consumption. Mathematically, the expected daily probability of alcohol consumption for individual *i* is

$$A_i = \frac{exp(\beta_1 x_i)}{7K}$$

where β_1 is the vector of coefficients to represent the effects of the variables in Table S2 (including the constant term), x_i is the vector of characteristics of individual *i*, and *K* is the adjustment for reporting bias (0.83, i.e. assuming 17% under-reporting of the frequency of alcohol consumption). The factor of 7 appears in the denominator because the regression model in Table S2 gives the number of drinking days per week, and we wish to convert this to a daily probability. We assume for simplicity that there is no alcohol consumption at ages younger than 15, although infrequent drinking at these younger ages is reported [40].

To this expected daily probability we add a random term a_i, to account for inter-individual variation in the frequency of drinking, which is not accounted for by the known covariates in Table S2. In the interests of simplicity, this random term is assumed to remain unchanged over time, for any given individual. The variance of this term is estimated by comparing the observed variation in the number of days on which alcohol was consumed and the variation predicted by the model (Table S4). This variance is inflated by an arbitrary factor of 1.2^2, to correct for the additional variance that is due to social desirability bias (i.e. different people understate their true level of alcohol use to different degrees). We thus assume that the standard error of the a_i terms is 0.29 in males ($1.973^{0.5} \times 1.2/7K$)) and 0.17 in females. The $A_i + a_i$ values assigned to each individual are truncated at 0 if less than 0, or 1 if greater than 1.

Table S4: Variation in numbers of days on which alcohol is consumed and numbers of drinks per drinking day

	Number of drinking days per week		Number of drinks per drinking day	
	Males	Females	Males	Females
Mean (self-reported)	0.761	0.210	6.59	4.04
Total variance	2.055	0.689	28.23	14.99
Variance explained by model	0.082	0.027	2.66	1.79
Residual variance	1.973	0.663	25.57	13.20

Source: Calculations from Tables S2 and Table S3, based on 2016 DHS data

We adopt a similar approach in using the regression model in Table S3 to assign to each individual an expected number of drinks per drinking day, B_i, except that this quantity is further adjusted to take into account the effects of personality and inequitable gender norms. Mathematically,

$$B_i = \frac{exp(\beta_2 x_i) + \gamma y_i + \delta(G_i - 0.2)I(g_i)}{K}$$

where β_2 is the vector of coefficients to represent the effects of the variables in Table S3, γ is the effect of conscientiousness on the amount of alcohol consumed per drinking day, y_i is the individual conscientiousness score, δ is the effect of endorsing inequitable gender norms on the amount of alcohol consumed per drinking day, G_i is the individual's probability of endorsing

inequitable gender norms (as defined in section 1.2), and $I(g_i)$ is an indicator of whether the individual's sex (g_i) is male ($I(g_i) = 1$) or female ($I(g_i) = 0$). The 0.2 in the above equation is the average inequitable gender norm score across all men.

The γ term has been set to -0.32, based on a simulation of the association between binge drinking (defined here as 5 or more drinks on a single occasion) and the conscientiousness score (in standard deviations from the population average); with $\gamma = -0.32$ the odds of consuming 5 or more drinks on a single drinking day reduces by 0.89 per standard deviation increase in the conscientiousness score, consistent with the meta-analysis of Hakulinen *et al* [5].

The δ term is set based on a South African study of the association between inequitable gender norms and alcohol abuse in young men, which found that each standard deviation increase in gender-inequitable norms score was associated with a 1.40-fold increase in the odds of alcohol abuse (95% CI 1.04-1.87) [19]. We set δ to 8.33, based on a simulation of the association between inequitable gender norms and binge drinking; with this parameter value, the odds of consuming 5 of more drinks on a single drinking day increases by 1.39 for each standard deviation increase in the gender-inequitable norm score, consistent with the findings of the South African study [19]. To represent the uncertainty in the parameter, we assign a gamma prior with a mean of 8.33 and a standard deviation of 4.

Similar to the approach described previously, we add to B_i a random b_i term to account for variation between individuals that is not explained either by the statistical model in Table S3 or the personality and gender norm effects described previously. The standard deviation of the b_i term is 7.31 in men ($25.57^{0.5} \times 1.2/K$) and 5.25 in women. The simulated value of $B_i + b_i$ is truncated at 1 if less than 1 (since a drinking day would not be classified as a drinking day if there was not at least one drink consumed).

In our model we allow for a direct effect of binge drinking on sexual risk behaviour (described in later sections), but we also make provision for associations between binge drinking and high-risk behaviour that are due to confounding factors not included in our model. For example, we do not model religiosity, which is negatively associated with both alcohol consumption and high-risk sexual behaviour [52]. Although we model directly the confounding effect of conscientiousness, which is negatively associated with both binge drinking and sexual risk behaviour, we do not model other dimensions of personality, such as extraversion and agreeableness, which are both likely confounders in the observed relationship between alcohol and sexual risk behaviour [5, 7]. Reverse causality may also be a source of 'confounding' (i.e. people may go to drinking venues to meet new sexual partners [53], rather than engaging in sexual risk behaviour because they go to drinking venues). To model this confounding, we allow for correlation between the simulated a_i and b_i terms and the individual's risk group. If R_1 is the random uniform variate used to assign the individual a risk group, and R_2 and R_3 are other random uniform variates, we assign the individual a_i and b_i terms using the value R_1 with probability p (where p can be considered the extent of the confounding in the observed association between alcohol and high-risk sexual behaviour) and using the values R_2 and R_3 (for a_i and b_i respectively) with probability $1 - p$. We assign a uniform (0, 1) prior to represent the uncertainty regarding the parameter p.

1.3.4 Model calibration

Figure S2 shows the model estimates of the average daily alcohol consumption (drinks per day) compared against estimates derived from alcohol sales data. The latter are derived from the Global Information System on Alcohol and Health, which includes estimates of unrecorded consumption (https://www.who.int/data/gho/data/themes/global-information-system-on-alcohol-and-health). The estimates, published as litres of pure alcohol per individual aged 15+ per year, are converted to a daily number of drinks on the assumption of 10g alcohol per 12.67 ml of pure alcohol, 20% wastage and 12g alcohol per standard drink [34, 47]. The model estimates of average consumption appear roughly consistent with the sales data.

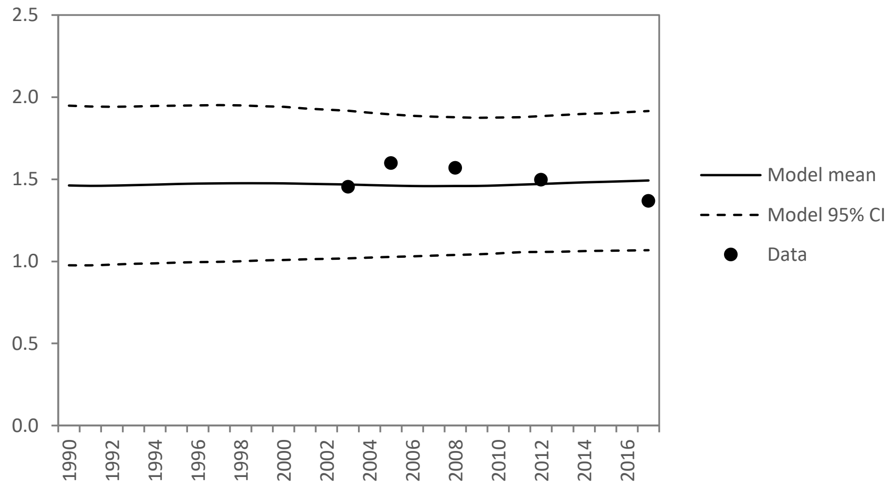

Figure S2: Average drinks per day, for individuals aged 15 and older

Figure S3 compares the model estimates of the proportion of adults (ages 15 and older) who have consumed any alcohol in the last month (defined as a daily drinking probability ≥ 1/30) against the results from 6 nationally-representative surveys [39, 41, 54-57], all of which asked questions about alcohol consumption in the last month or in the last year. Because we expect this self-reported measure of alcohol use to be understated, we compare the model estimates with survey estimates that are adjusted for under-reporting. The adjusted estimates are calculated assuming the ratio of the odds of alcohol use to the odds of *reported* condom use is 2 in men and 5 in women (i.e. roughly consistent with the odds ratios noted previously, but assuming greater under-reporting in women than in men). With these assumptions about reporting bias, the adjusted model estimates appear roughly consistent with the survey data.

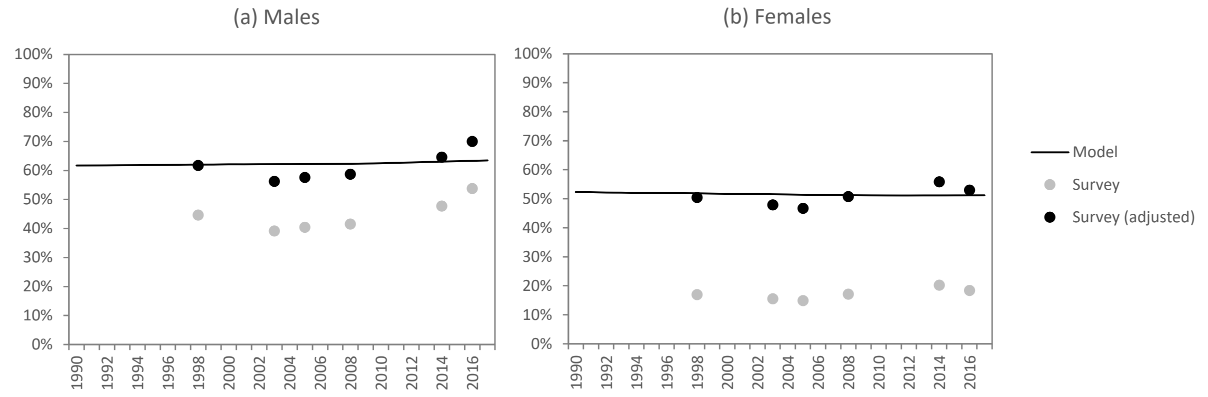

Figure S3: Proportion of adults aged 15 and older who consume alcohol at least monthly
Adjusted survey estimates are calculated on the assumption that the odds of alcohol use is 2 times the odds of reporting alcohol use in men, and 5 times the odds of reporting alcohol use in women.

Figure S4 compares the model estimate of the proportion of youth who engage in binge drinking (defined here as at least 5 drinks on a single drinking day) on at least a monthly basis, against the results from the 2002 Youth Risk Behaviour Survey (conducted among learners in grades 8-11) [58], the 2008 Youth Risk Behaviour Survey [40], and the 2011 Youth Risk Behaviour Survey [59]. All surveys relied on self-completed questionnaires, and are therefore less likely to be affected by the social desirability biases described previously. Nevertheless, the model estimates appear slightly higher than the Youth Risk Behaviour Survey data, suggesting that there may still be some reporting bias in self-completed questionnaires. It is also possible that there may be non-response bias (for example, in the 2008 and 2011 surveys the response rate was 72%, which could bias the results if the youth who were not surveyed were more likely to be binge drinkers).

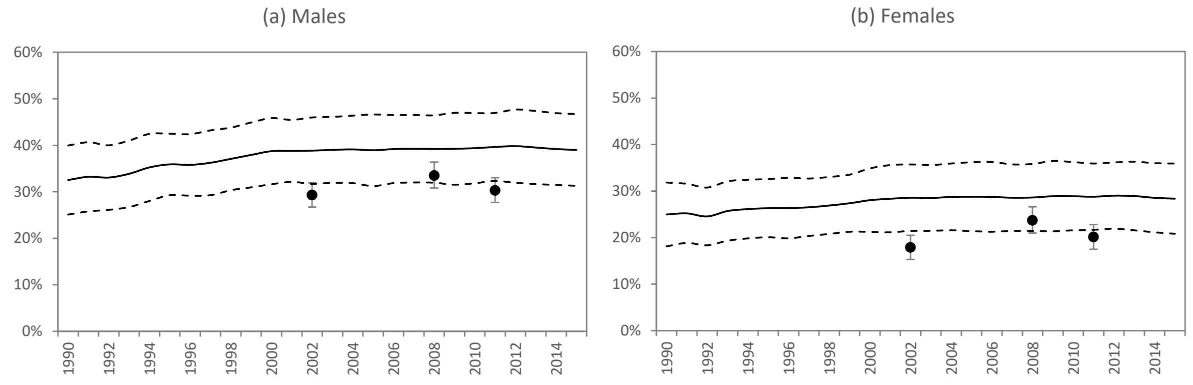

Figure S4: Proportion of high school students (grades 8-11) who have had 5 or more drinks on a single day in the last month
Dots represent self-reported measures in national surveys. Solid lines represent the average of the model results and dashed lines represent 95% confidence intervals.

1.3.5 Comparison with South African Medical Research Council estimates

The South African Medical Research Council (SAMRC) has recently developed a Bayesian approach to estimating levels of alcohol consumption in South Africa from household survey data [60, 61]. Similar to our method, the SAMRC model includes adjustment factors to correct for under-reporting of alcohol consumption in surveys, and both models aim to match estimates

derived from alcohol sales data. However, the SAMRC approach differs from ours in several respects:

- We have relied on a single survey (the 2016 DHS) in setting our model parameters, whereas the SAMRC combines data from 17 national surveys (between 1998 and 2016). Although the inclusion of more data is a strength, it is worth noting that the estimated bias due to under-reporting is much smaller in the 2016 DHS than in the other surveys [60].
- Because of the inclusion of multiple surveys, the SAMRC model allows for changes in alcohol consumption over time (whereas our model assumes that after controlling for age, sex and other socio-demographic factors, alcohol consumption levels remain fixed over time). Overall, the SAMRC model suggests a modest decline in alcohol consumption per capita over 1998-2016 (by 21% in men and 15% in women), though with substantial variation across age groups.
- The SAMRC model does not account for factors such as socio-economic status and urban-rural location in modelling alcohol consumption (see Tables S2-S3).
- The SAMRC model splits the population into people who never drink and people who do drink, and then estimates an average number of drinks per day among the latter, fitting a gamma distribution to represent inter-individual variation in drinks per day (among those who drink). In our model, however, we decompose the average drinks per day into the proportion of days on which alcohol is consumed, and the average number of drinks per drinking day. This decomposition is important in distinguishing between (for example) infrequent binge drinking and regular drinking of small quantities of alcohol.

Our estimates of the ‘prevalence of drinking’ (proportion of people who consume any alcohol in a month) are reasonably consistent with the SAMRC estimates in men, but not in women. In the latter, for example, we estimate the prevalence of drinking to be around 50% (Figure S3), while the SAMRC estimates a prevalence of around 20%. The 20% prevalence estimate is very close to the self-reported prevalence estimates in surveys (grey dots in Figure S3b), suggesting that the SAMRC model makes little or no adjustment for social desirability bias in the self-reported prevalence data. However, as we noted in our earlier discussion, studies suggest that there is substantial under-reporting of the prevalence of drinking in African settings, especially in women [44, 45]. Our model is validated using data from the South African Youth Risk Behaviour Surveys, which rely on more anonymous interviewing methods than the surveys included in the SAMRC model (Figure S4). We would not be able to match the high levels of binge drinking reported by adolescent girls in these surveys if we did not make provision for substantial under-reporting of the prevalence of drinking in other surveys that rely on face-to-face interviews.

1.4 Modelling concurrent partnerships

In our model, only ‘high-risk’ individuals enter into concurrent partnerships. For men in the high-risk group, the relative rate at which secondary partners are acquired (relative to the rate at which primary partners are acquired) is $\Omega_l \Psi_i(r)$, where Ω_l is an adjustment for the relationship type (0.64 for men in non-marital relationships (l = 0) and 0.41 for men in marital relationships (l = 1)) and

$$\Psi_i(r) = k_r\big(1 + \theta(G_i - 0.2)\big)$$

where k_r is a race-specific constant term, G_i is the gender-inequitable norm score for individual i, as defined in section 1.2, and θ is the increase in the rate of entry into concurrent partnerships when comparing men who always endorse inequitable gender norms to that in men who never endorse inequitable gender norms (we assume $\Omega_l\ \Psi_i(r)$ is bounded on the range [0, 1]). The 0.2 in the above equation is the average gender-inequitable norm score in men (as noted in section 1.2), so that the gender-inequitable norm adjustment is equal to one in men who have average levels of inequitable norm endorsement. As described previously [1], the k_r terms are set by calibrating the model to data on relative rates of reporting of concurrent partnerships, by race.

To estimate θ, we rely on a study of correlates of inequitable gender norms in young men in rural Mpumalanga province (South Africa) [19]. In this study, each standard deviation increase in the gender-inequitable norm score was associated with a 1.31-fold increase (95% CI: 1.07-1.62) in the odds of reporting concurrent partnerships (controlling for age, education and other factors). In our model, the standard deviation of gender-inequitable norm scores is approximately 0.12, which suggests the value of θ should be around 2.6 ((1.31 – 1)/0.12). Substituting the lower and upper limits of the 95% confidence interval into the above equation gives values of 0.6 and 5.2 respectively. However, these estimates may be under-estimates of the true θ because a more pronounced effect on the rate of entry into concurrent partnerships is required to match an observed effect that is expressed in terms of the *cumulative* probability of concurrency. To see this, suppose that we are comparing high-risk men with inequitable norm scores of 0.3 and 0.2. If $\theta = 2.6$, the ratio of $\Psi_i(r)$ values would be 1.26. But if the annual rate of entry into concurrency in high-risk men was 1.2 per annum when $G_i = 0.2$, the ratio of the cumulative probability of any concurrency over one year would be only 1.12 ((1 – exp(-1.2 × 1.26))/ (1 – exp(-1.2)). In this example, the 'true' effect (a 26% difference in the rate of entry into concurrent partnerships) is more than double the 'observed' effect (a 12% difference in the cumulative probability of concurrent partnerships). To represent the uncertainty in the θ parameter, we therefore use a gamma prior with a mean of 5 and a standard deviation of 2.5 (which has 2.5 and 97.5 percentiles at 1.4 and 11.0 respectively).

For women in the high-risk group, the same approach is taken to modelling the rate of entry into concurrent relationships, except that the adjustments for the current relationship (Ω_l) are lower (0.54 for women in non-marital relationships and 0.17 for women in marital relationships), and the adjustment for gender-inequitable norm score is not included (since we do not model gender-inequitable norms in women).

1.5 Modelling casual sex

Previous versions of MicroCOSM have not explicitly included transactional sex, which appears to be a key pathway linking socio-economic status and HIV risk behaviour. However, the term 'transactional sex' is difficult to define [62] and the extent to which sex is transactional might be more appropriately considered as lying on a spectrum [62, 63]. Rather than model transactional sex, we introduce a new relationship category, casual once-off sexual relationships ('casual sex' for short), and assume that both male and female engagement in casual sex is linked to their socio-economic status (recognizing that in the South African setting, casual sex is often transactional [64, 65]). The model already includes casual sex for men who have sex with men (MSM) [66], and we model heterosexual casual sex using a similar approach: individuals are assumed to move in and out of casual sex 'phases' depending on their age, risk group, relationship status, socio-economic status and level of binge drinking. We define casual sex relationships as relationships that last less than a week (mostly involving a single sex act). To be consistent with the modelling of casual sex in MSM (and because we

lack data on the assortativeness of sexual mixing in the casual sex context), we assume that heterosexual casual sex mixing is random with respect to age, educational attainment and risk group, but completely assortative with respect to race (i.e. individuals choose casual sex partners from their own race group). As in the MSM model, we assume that individuals remain in the casual sex state for a year on average, after entry (this assumption is not important as it is the rate of *entry* into the casual sex state that principally determines the overall prevalence of casual sex).

Although a few South African studies have investigated levels of ‘casual sex’, relatively few have specifically measured the incidence of once-off sex acts, which corresponds more closely with our model definition of casual sex. One study among medical students in Pretoria found that 28% of males reported having had ‘one-night stands’ in the last 6 months, but the proportion was only 4% in females [67]. Another study in Cape Town, conducted mainly in African men aged 25-44, all of whom reported at least two sexual partners in the last 3 months, found that 94% reported concurrent sexual partners in the last 3 months, 71% reported at least one once-off partner, and there were on average 5 sexual partners in the last 3 months [65]. We have calibrated the model assumptions about the male rate of entry into casual sex in the baseline category (African men aged 20, who are high-risk and single) such that the model output (% of African men aged 25-44 with two or more partners in the last 2 months, who have had once-off sex partners) is consistent with the 71% observed in this study, and have set the assumed frequency of casual sex acts during the casual sex state (Table S5.1) such that the model matches the reported average of 5 sexual partners in this group. We further assume that the relative rate of entry into the casual sex state for low-risk men is 0.4, in order to match (approximately) the 94% reporting of concurrent partnerships in men who have recently had multiple partners (our model definition of low-risk excludes men who engage in concurrent partnerships). Another study among pregnant African women in Soweto found that 29% reported having ever engaged in a once-off sexual partnership, and 66% of the women who reported once-off sexual encounters also reported having ever engaged in a concurrent relationship [68]. We have therefore set the female rates of entry into casual sex such that the modelled proportion of pregnant African women who have ever had a once-off partner is 29%, and the relative rate of entry into casual sex in low-risk women is set to 0.3, such that the modelled fraction of pregnant African women with a history of casual sex who are high risk is close to 66%. In the interests of simplicity, we assume relative rates of entry into casual sex by age are the same as for short-term relationships. High-risk individuals who are already in relationships are assumed to have a rate of entry into the casual sex state that is 0.8 times that assumed for single high-risk individuals. However, low-risk individuals who are already in relationships are assumed not to enter the casual sex state (since we define ‘low-risk’ as not engaging in concurrent relationships). Similarly, if a low-risk individual is in the casual sex state but they acquire a regular (short-term) partner, we assume that they immediately leave the casual sex state.

Table S5.1: Model assumptions about heterosexual rates of entry into the casual sex state

Parameter	Male	Female
Annual rate of entry in high-risk unpartnered individuals aged 17.5	0.3*	0.2
Relative rate of entry in unpartnered low-risk individuals	0.4	0.3
Relative rate of entry in partnered high-risk individuals	0.8	0.8
Desired monthly frequency of casual sex	10	10

* In men who have a propensity for same-sex relationships, this rate is reduced in proportion to their preference for male partners.

Table S5.2 shows some of the key model casual sex outputs for 2005, and compares these against the calibration targets mentioned previously. It is worth noting that it is difficult to match both the frequency of casual sex in men and the proportion of men who have recently engaged in casual sex: increasing the proportion of men who engage in casual sex (but not the proportion of women who engage in casual sex) leads to a reduction in the number of sexual partners per man engaging in casual sex because there are relatively few women available to engage in casual sex. The discrepancy between the male demand for casual sex relationships and the female demand for casual sex relationships also implies that the desired monthly frequency for casual sex in men (which applies only to the men who are assigned casual sex partners) exceeds the actual average male frequency of casual sex quite substantially.

Table S5.2: Heterosexual casual sex model outputs in 2005

Model output	Value	Calibration target
% of 15-49 year old men engaging in heterosexual casual sex	14%	-
% of 15-49 year old women engaging in casual sex	4%	-
% of men with multiple partners who have had casual sex in the last 3 months	69%	71%
Average # partners in the last 3 months, for men who report multiple partners in the last 3 months	4	5
% of men with multiple partners in the last 3 months who are 'high-risk'	93%	94%
% of pregnant women who have ever had casual sex	30%	29%
% of pregnant women reporting a history of casual sex who also report a history of concurrent partnerships	64%	66%

In our modelling of condom use by MSM, we previously assumed that the odds of condom use in casual sex encounters was 2.2 times that in regular non-cohabiting relationships, based on South African MSM data [69]. This is quite consistent with DHS survey data for men in the 'general population' in other African settings: Adair [70] found that in five sub-Saharan African countries, the odds of condom use with a casual partner was consistently 1.5-2.8 times that in regular non-marital relationships. We therefore use the same multiple of 2.2 to model the relative odds of condom use in casual heterosexual sex acts.

1.5.1 The effect of binge drinking on entry into casual sex

Several observational studies in South Africa have found strong associations between binge drinking and casual/transactional sex. These associations are especially strong in women, with observed odds ratios for the association between recent binge drinking and transactional sex lying in the range 2.0-5.4 (average value of 3.3) [49, 68, 71, 72]. Associations are more modest in men, ranging between 1.3 and 2.6 (average value of 1.8) [49, 65, 73, 74]. A randomized controlled trial in Cape Town found that alcohol counselling interventions led to substantial (though not quite significant) reductions in the reporting of meeting partners in shebeens (which might be considered a proxy for casual sex) [75], and another randomized trial in the Western Cape found that alcohol counselling led to a slight (but not significant) reduction in reporting of casual sex 12 months after baseline [76].

In our model, we assume that the rate of entry into casual sex relationships increases by a factor of $(1 + A_g)$ in individuals of sex g, if they engage in binge drinking at least once a month. To represent the uncertainty in the A_1 parameter (in men), we assign a gamma hurdle distribution:

with probability 0.5, a value of 0 is assigned, and with probability 0.5 we sample from a gamma distribution with mean 0.75 and standard deviation 0.3 (consistent with the observational studies cited in the previous paragraph). Similarly, we represent the uncertainty in A_2 (in women) using a gamma hurdle distribution with the same probability weights but with mean and standard deviation of 2.3 and 1.0 respectively for the gamma distribution. The inclusion of the 'hurdle' at zero allows for the possibility that the observed associations between binge drinking and casual sex might be explained entirely by confounding factors, and might not represent a true effect of binge drinking on casual sex.

1.5.2 The effect of inequitable gender norms on men's entry into casual sex

Observational studies from South Africa, Ghana and Tanzania have found associations between men's endorsement of inequitable gender norms and their reporting of transactional sex [22, 77]. A randomized controlled trial of a gender transformative intervention in South Africa was found to significantly reduce men's reporting of transactional sex with casual partners over one year, but the overall reduction in casual partners was not statistically significant [32]. Another South African randomized trial of a similar intervention also found a modest (but not significant) reduction in men's reporting of transactional sex with casual partners [30]. There is thus some evidence to suggest that inequitable gender norms may have an effect on men's entry into casual sex.

Similar to the approach described in section 1.4, we assume that inequitable gender norms affect high-risk men's rates of entry into the casual sex state. The relative rate of entry into casual sex for a man with inequitable norm score G_i is calculated as

$$\beta^{((0.2-G_i)/0.1)}$$

where β is the factor by which the rate of casual sex entry reduces per 0.1 decrease in the inequitable norm score, and 0.2 is the average inequitable norm score. We represent the uncertainty in this parameter using a beta hurdle distribution: with probability 0.5 a value of 1 is assigned (i.e. assuming all of the observed association between inequitable gender norms and casual/transactional sex is due to confounding), and with probability 0.5 a parameter is sampled from a beta distribution with mean 0.75 and standard deviation 0.2.

1.5.3 The effect of socio-economic status on entry into casual sex

South African studies suggest that employed men are more likely to engage in transactional sex with casual partners than unemployed men [74], and that transactional sex is marginally associated with wealth and employment status [56]. We assume that the previously specified base rate of male entry into casual sex applies to unemployed men, and that this factor is increased by a factor of 1.5 in employed men.

In contrast, socioeconomic status in South African women appears to be negatively associated with engagement in casual/transactional sex. In a study of women attending drinking venues in Cape Town, the odds of recent transactional sex was 2.0 and 3.6 time greater in women living in houses with no electricity and no running water respectively, although individual educational attainment and employment status did not predict transactional sex [71]. Similarly, in a large survey of adolescent girls and young women in South Africa, reporting of transactional sex was strongly associated with household food insecurity [78]. In a study in Soweto, women who reported living in 'substandard housing' had a 1.7-fold increased odds of

transactional sex, but individual-level socioeconomic status was inconsistently related to transactional sex (higher education was negatively associated but employment was positively related) [68]. These studies suggest it is women's *household* socioeconomic status that determines their engagement in transactional sex, and the role of individual socioeconomic status is less clear. We therefore assume that for each unit decrease in per capita household income (on a log scale), there is a 50% increase in women's rate of entry into the casual sex state. (The base rate of entry into casual sex specified previously applies at the average log per capita household income.)

1.6 Modelling condom use

The model of condom use is the same as described previously [1], but has been modified to take into account the effect of educational attainment, household income, binge drinking and inequitable gender norms on condom use.

1.6.1 The effect of education on condom use

Suppose $\gamma(h,t)$ is the probability of condom use in short-term (non-cohabiting relationships) in year t, among individuals of educational attainment h. In a simple model that ignores the effect of educational attainment on condom use, we might model the time trend in condom use using the equation

$$\log\left(\frac{\gamma(h,t)}{1-\gamma(h,t)}\right) = \kappa_1 + (\kappa_2 - \kappa_1)\left(1 - 0.5^{(t/M)^Q}\right)$$

where κ_1 and κ_2 are initial and 'ultimate' levels of condom use respectively, and the transition between these initial and ultimate condom levels is modelled using a cumulative Weibull distribution with shape parameter Q and median M (t and M are both measured in years after 1985). This is similar to the model that we used previously to model trends in condom use in South Africa [1, 79].

There are several possible ways in which education effects can be incorporated into this model. The simplest approach is to assume that the effect of educational attainment remains fixed over time. For example, we might assume that

$$\log\left(\frac{\gamma(h,t)}{1-\gamma(h,t)}\right) = \kappa_1 + (\kappa_2 - \kappa_1)\left(1 - 0.5^{(t/M)^Q}\right) + \beta(h - 10)$$

where h is the number of years of completed education, and $\exp(\beta)$ represents the increase in the odds of condom use per year of completed schooling. This is similar to the model of educational effects that was previously assumed in MicroCOSM version 2.0, except that the earlier model treated the education effect as a categorical variable rather than a continuous variable [1]. We previously relied on data from the 1998 and 2003 DHSs to determine the education effects. Using the same data here, we estimate plausible values of the β parameter to be around 0.16 (note that 'completed tertiary education' is treated as h = 15 for the purpose of estimating this model).

A limitation of this model is that it does not allow for the possibility that the effect of education may be changing over time. 'Diffusion of innovations' theory posits that a subset of the population are early adopters of a new health intervention, and the rest of the population

eventually follow their example [80]. To the extent that the early adopters are more likely to be well-educated individuals of higher socio-economic status [80], one might expect to see a larger socio-economic difference in the adoption of the new intervention early on than later, once the intervention has been widely adopted. Indeed, evidence of such attenuations in the socio-economic gradient of condom use have been found in countries such as Burkina Faso [70] and Brazil [81]. Thus an alternative model, more in line with 'Diffusion of innovations' theory, would be one in which the education effect applies to the median parameter:

$$\log\left(\frac{\gamma(h,t)}{1-\gamma(h,t)}\right) = \kappa_1 + (\kappa_2 - \kappa_1)\left(1 - 0.5^{\left(t/(M\alpha^{h-10})\right)^Q}\right).$$

Here α is the factor by which the median time to adoption reduces per year of additional education. We can roughly estimate the α parameter from the study of Adair [70], who analysed DHS data on men's condom use in non-marital relationships in five different African countries, in each case comparing the effect of educational attainment on condom use in DHSs conducted approximately five years apart. In countries such as Cameroon, in which there was a low initial rate of condom use, the socioeconomic gradient increased over the 5-year period, while in countries such as Burkina Faso, with a high initial condom use, the socioeconomic gradient declined. Figure S5 shows the results obtained from fitting the above model to the data from the Adair study: approximate consistency is achieved when we set α = 0.96.

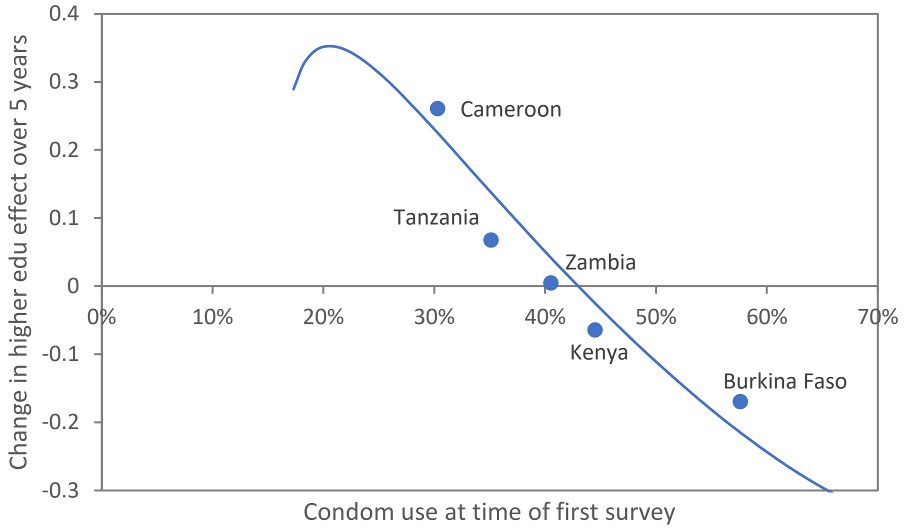

Figure S5: Changes in the relationship between educational attainment and condom use over time

Dots represent estimates based on data presented by Adair [70]. The change in higher education effect (y axis) is the change in the coefficient from an ordinal logistic regression model, on a logit scale, that relates condom use at last sex to an educational attainment categorical variable (with 4 levels), the change being assessed between successive DHSs, five years apart. For the purpose of fitting a model (solid line), we set κ_1 and κ_2 to -1.66 and 2.20 respectively (based on the data presented by Adair [70]), and Q = 3.25 and M = 14 (based on values previously estimated in fitting the MicroCOSM model [1]). For the purpose of estimating α, we assume each change in education category (from the ordinal logistic regression model) is equivalent to a 3-year change in completed years of schooling.

In our revised model, we allow for both types of adjustment, i.e. we set

$$\log\left(\frac{\gamma(h,t)}{1-\gamma(h,t)}\right) = \kappa_1 + (\kappa_2 - \kappa_1)\left(1 - 0.5^{\left(t/(M\alpha^{h-10})\right)^Q}\right) + \beta(h-10).$$

However, in calibrating the model to match observed associations between completed secondary education and condom use, it was necessary to change the values of α and β to 0.93 and 0.05 respectively.

1.6.2 The effect of household income on condom use

South African studies have found an association between higher household income and higher rates of condom use [82, 83]. Although this could be due to confounding with educational attainment (discussed in the previous section), randomized trials of cash transfers and microfinance interventions [84-86] have in some cases been found to lead to significant increases in condom use [86]. We assume that for each unit increase in the log of the per capita household income, the odds of condom use increases by a factor of 1.04 [82].

1.6.3 The effect of binge drinking on condom use

South African studies show strong associations between binge drinking and unprotected sex. A study of individuals attending drinking venues in Cape Town found that for each unit increase in the first two AUDIT responses (relating to the frequency and quantity of alcohol consumed), there was a roughly 20% increase in the frequency of unprotected sex, with the results being similar in men and women [87]. A similar study in North West province found the frequency of unprotected sex increased by 21% in men and by 29% in women, per unit increase in AUDIT score [50]. Other South African studies involving structural equation modelling have found strong associations between levels of alcohol consumption and frequencies of unprotected sex [88, 89]. A number of randomized controlled trials of alcohol counselling interventions in sub-Saharan Africa have found significant reductions in unprotected sex, suggesting a causal relationship between alcohol consumption and condom use [75, 90, 91], although some randomized trials have not found any significant effect of alcohol counselling interventions on unprotected sex [76, 92].

In our model we assume that the log odds of condom use (as defined in section 1.6.1) reduces by a factor of λ for each additional day of binge drinking per week. This implies that people who never engage in binge drinking will not reduce their frequency of condom use, and that people who do engage in binge drinking will have a reduced rate of condom use as their frequency of binge drinking increases. The value of λ can be crudely estimated from the previously cited studies. For example, consider two individuals, one who engages in binge drinking once per week and another who engages in binge drinking twice per week (in every other respect the two individuals have identical drinking behaviours). Using the first two AUDIT questions, we would expect a 1 unit difference between the individuals in their summed responses, and based on the Cape Town study [87] we would expect this to represent a 20% increased rate of unprotected sex for the second individual, or equivalently, $\lambda \approx 0.83$ (1/1.2). However, in our model actual condom use depends on both the individual's characteristics and their partner's characteristics – we average the two partners' desired condom preferences when determining whether a condom is used. If the two individuals in our example had partners with the same condom preferences, we would need to set $\lambda \approx 0.65$ in order to reproduce the observed relative risk of 1.2 (since $1/(0.5 \times (1 + 0.65)) \approx 1.2$, assuming both partners have the same condom preference as the first individual). It is possible to construct other examples in which λ is more or less than 0.65, and this is not intended to be an exact justification. To represent the

prior uncertainty in λ, we assign a beta hurdle distribution: with probability 0.5 we set $\lambda = 1$ (i.e. there is a 50% chance that the observed association between alcohol and condomless sex is due to confounding), otherwise we sample λ from a beta distribution with mean 0.65 and standard deviation 0.2.

1.6.4 The effect of inequitable gender norms on men's condom use

South African studies have found associations between inequitable gender norms and men's levels of condom use. In men living with HIV in KwaZulu-Natal, Fladseth *et al* [24] found that men with above-average equitable norm scores had an odds of condom use that was 2.3 times (95% CI: 1.2-4.5) that in men with below-average scores, although this odds ratio reduced to 1.9 (95% CI: 0.9-4.1) when controlling for other factors. Gibbs *et al* [89] also found, in young men in South African informal settlements, that each standard deviation increase in inequitable gender norms was associated with a roughly 7% reduction in men's reporting of condom use in a structural equation model. One randomized trial of a gender-transformative intervention in Uganda found a significant increase in men's reporting of condom use [93]. Other randomized trials of gender-transformative interventions in South Africa have found short-term increases in men's condom use, although these increases have not been statistically significant [29, 32].

In our model we assume that the odds of condom use are reduced by a factor of δ in men who always endorse inequitable gender norms, when compared to men who never endorse inequitable gender norms. Mathematically, the odds of condom use in individual i are multiplied by $\delta^{G_i-0.2}$, where G_i is the gender inequitable norm score for individual i, and 0.2 is (roughly) the average gender inequitable norm score. To represent the prior uncertainty in δ, we assign a beta hurdle distribution: with probability 0.5 we set $\delta = 1$ (i.e. there is a 50% chance that the observed association between inequitable gender norms and condomless sex is due to confounding), otherwise we sample δ from a beta distribution with mean 0.25 and standard deviation 0.15. The standard deviation of gender inequitable norm scores in our model is around 0.12, and the value of 0.25 is therefore in between the values we might expect based on the Fladseth study ($0.04 = (1/1.9)^{1/\left(0.12\times 2\times\sqrt{2/\pi}\right)}$) and the Gibbs study ($0.55 = (1 - 0.07)^{(1/0.12)}$).

1.7 Modelling alcohol counselling interventions

Systematic reviews of alcohol counselling interventions have shown that single-session counselling interventions (also known as "brief alcohol interventions") are less effective than multi-session alcohol counselling interventions [94]. We therefore consider separately single-session and multi-session counselling interventions. Randomized trials of these interventions in sub-Saharan Africa generally find that for both single-session and multi-session interventions, the effect of the counselling is greater in the short term than in the longer term [75, 90, 91]. We therefore assume that the relative frequency of alcohol consumption (in terms of the number of drinking days per week) at time t after the start of intervention i (1 for single-session, 2 for multi-session counselling) is of the form

$$R_{it} = 1 - (1 - B_i)(1 - A)^t$$

where B_i is the relative frequency of alcohol consumption immediately after the start of intervention i, and A is the proportionate reduction in the intervention effect per year. We use

a slightly different adjustment for the relative quantity of alcohol consumed (in terms of the numbers of drinks per drinking day):

$$S_{it} = 1 - \left(1 - \sqrt{B_i}\right)(1 - A)^t.$$

We use $\sqrt{B_i}$ because data from African RCTs suggest a greater impact of alcohol counselling interventions on the frequency of drinking than on the number of drinks per drinking day [95], and this approach avoids introducing additional parameters. It is the product of these two adjustments that determines the relative volume of alcohol consumed per week (so that at time 0, immediately after the delivery of the alcohol intervention, the relative volume of alcohol consumed is $R_{i0}S_{i0} = B_i^{1.5}$). It can be shown that B_i is approximately equal to the relative probability of binge drinking,[1] which is advantageous for the purpose of estimating B_i. We assign prior distributions to represent the uncertainty in each of these parameters. Our approach is to set the priors based on international reviews and trials, and not on trials in sub-Saharan Africa, in order to avoid using the same data in setting the prior distributions and in defining the likelihood function.

In the case of the effectiveness of single-session counselling interventions, a recent meta-analysis, limited to trials in high-income settings, found the odds of binge drinking reduced by a factor of 0.67 on average (95% CI: 0.58-0.77) [94]. Although this outcome was not reported separately for single-session and multi-session interventions, the review found that the proportionate reduction in alcohol consumption was approximately 2.5 times greater in the latter than in the former. There were roughly equal numbers of single-session and multi-session interventions in the meta-analysis, and thus one might expect that $0.67 \approx \sqrt{B_1 B_2} = \sqrt{B_1 B_1^{2.5}} = B_1^{1.75}$. This suggests $B_1 \approx 0.80$ and $B_2 \approx 0.56$. However, there is significant uncertainty around these estimates. Some have argued that earlier meta-analyses were affected by publication bias, which led to intervention effects being exaggerated [96]. On the other hand, meta-analyses have focused on levels of alcohol consumption 6-12 months after baseline, which could lead to the short-term effect of the intervention (immediately after the counselling) being under-estimated, due to the previously noted attenuation of the intervention effect at longer durations. The meta-analyses could also under-estimate effectiveness because many of the interventions included in the review did not involve ‘direct’ person-to-person contact (e.g.

[1] Suppose that G represents the relative probability of binge drinking immediately after the intervention compared to the period before the intervention (where the outcome is binge drinking at least once a month). We can decompose this into the relative probability of drinking at least once a month after the intervention (r_1) and the relative probability of drinking at least 5 drinks per drinking day after the intervention (r_2), i.e. $G = r_1 r_2$. Suppose X, the number of drinking days per month among people who were binge drinkers at baseline, is exponentially distributed with parameter π. (By definition, $X \geq 1$ in order for people to be classified as binge drinkers in our model.) Then $Pr[XB_i < 1 | X \geq 1] = \int_1^{1/B_i} \pi e^{-\pi x} dx / \int_1^{\infty} \pi e^{-\pi x} dx$. From this it follows that $r_1 = exp\left(-\pi\left(\frac{1}{B_i} - 1\right)\right)$. Our preliminary model parameterization suggests a value of π around $\frac{1}{3}$, and with these values we find $r_1 \approx \sqrt{B_i}$. Similarly, suppose Y, the number of drinks per drinking day among all people who are drinkers at baseline, is exponentially distributed with parameter λ. (By definition, $Y \geq 5$ in order for people to be classified as binge drinkers in our model.) Then following the same mathematical derivation as before, it follows that $r_2 = exp\left(-5\lambda\left(\frac{1}{\sqrt{B_i}} - 1\right)\right)$. Our preliminary model parameterization suggests a value of λ around $\frac{1}{5}$, and based on this we get $r_2 \approx \sqrt{B_i}$. Thus we get the result $G = r_1 r_2 \approx B_i$. For example, if $B_i = 0.65$, then using the above equations $r_1 = 0.836$, $r_2 = 0.786$, and $G = r_1 r_2 = 0.657$.

counselling was through an automated system or online tool), in contrast to the trials evaluated in sub-Saharan Africa [94]. Given the potential biases, we represent the uncertainty around B_1 by assigning a beta prior with a mean of 0.80 (i.e. equivalent to a roughly 20% reduction in the probability of binge drinking) and a standard deviation of 0.10. Similarly we represent the uncertainty around B_2 using a beta prior with a mean of 0.55 and a standard deviation of 0.20.

Data on the extent to which alcohol counselling effects attenuate over time are limited. Findings from the TrEAT trial suggest a roughly 50% reduction in the intervention effect between 12 and 48 months after the intervention [97], which suggests an A value of 0.21 ($1 – 0.5^{1/3}$). In an evaluation of the Preventure intervention in the UK, the quantity of alcohol in the intervention group was 12% less than that in the control group at 6 months but 5% less at 18 months [98], suggesting an A value of 0.58 (1 – 0.05/0.12). A brief alcohol intervention tested in Australia was found to significantly reduce alcohol consumption by 9 months, but by 10 years after the intervention, there was no difference between the intervention and control groups in levels of drinking [99]. Given the limited and inconsistent data, we assign a vague prior (uniform on the range [0, 1]) to represent the uncertainty in parameter A.

1.8 Modelling gender transformative interventions

A previous review of gender transformative interventions found that significant improvements in gender equitable norms occurred in several studies [100]. However, only 3 of the 15 studies were randomized controlled trials, which makes it difficult to determine the true effect of the interventions. Given the heterogeneity in study designs, the authors did not conduct any meta-analysis. More recent reviews of gender-transformative interventions have been similarly challenged by heterogeneity in outcomes and interventions, and have also not attempted meta-analysis [101-103].

In our model we distinguish between two types of gender-transformative interventions: those that aim to change gender norms through individual or group counselling and those that aim to change gender norms at a community level, typically through community mobilization [101]. Similar to the approach described in the previous section, we assume that for intervention type i, (1 for individual-based, 2 for community-based) the relative rate of endorsing inequitable gender norms in men who have just been exposed to the intervention is B_i, when compared to their levels prior to the intervention. Randomized trials of gender transformative interventions in sub-Saharan Africa typically find a much more significant short-term benefit than is observed in the longer term [30, 32]. We therefore assume, as with alcohol counselling interventions, that the reductions in inequitable gender norms wane over time. If R_{it} is the relative rate of endorsing inequitable norms t years after the intervention start, in men exposed to intervention type i, then

$$R_{it} = 1 - (1 - B_i)(1 - A)^t$$

where A is the proportionate reduction in the intervention effect per year. Given the uncertainty around the intervention effectiveness, we assign uniform (0, 1) priors to each of the A, B_1 and B_2 parameters.

2. Method for estimating confidence intervals

The calculation of uncertainty ranges for agent-based models is complex because variation in model outputs can be attributable both to variation in model parameters and stochastic variation in model outputs (i.e. even when model parameters are identical, results can differ across simulations due to differences in random numbers used to simulate outcomes). We are interested primarily in the former source of uncertainty, and by not adjusting for the stochastic variation we may potentially exaggerate the extent of the uncertainty associated with changes in model parameters.

Suppose we wish to estimate a proportion (between 0 and 1), based on 50 different simulations (each simulation being generated using a different combination of input parameters). In this study, the proportions in which we are interested are population attributable fractions (PAFs) and proportionate reductions in HIV/STI incidence due to different interventions. Because the proportion has to be on the range (0, 1), it would be reasonable to assume the 'true' distribution of proportions follows a beta distribution, i.e. the probability that a randomly chosen input parameter combinations generates a proportion p is

$$Beta(p|m,n) = p^{m-1}(1-p)^{n-1}\frac{\Gamma(m+n)}{\Gamma(m)\Gamma(n)}$$

where *m* and *n* are the parameters of the beta distribution.

Now suppose that for a given parameter combination, we run the model twice, with different random numbers, and obtain estimates x_1 and x_2 of the proportion we are trying to estimate. (It is important to run the model at least two times for each parameter combination, otherwise it is impossible to obtain an estimate of the stochastic variation.) Although the true proportions must lie on the interval (0, 1), the *observed* proportions can lie anywhere on the interval (-∞, 1). (This is because both the PAF and intervention impact outputs are calculated using the formula $1 - a/b$, where a and b represent positive-valued model outputs under two different scenarios.) Hence if we define $y_1 = 1 - x_1$ and $y_2 = 1 - x_2$, both y_1 and y_2 will lie on the range (0, ∞). We assume that for a given proportion *p*, the distribution of possible y_i values is gamma-distributed with mean $1 - p$ and shape parameter *α*, i.e. the probability of observing y_i is

$$Gamma(y_i|p,\alpha) = \left(\frac{\alpha}{1-p}\right)^{\alpha} {y_i}^{\alpha-1} exp\left(-\frac{\alpha y_i}{1-p}\right)\frac{1}{\Gamma(\alpha)}$$

and thus the joint distribution of y_1 and y_2 is

$$Gamma(y_1, y_2|p,\alpha) = \left(\frac{\alpha}{1-p}\right)^{2\alpha} (y_1 y_2)^{\alpha-1} exp\left(-\frac{\alpha(y_1+y_2)}{1-p}\right)\frac{1}{\Gamma(\alpha)^2}.$$

The likelihood, integrating across all possible values of *p*, is then

$$L(y_1, y_2|m,n,\alpha) = \int_0^1 Gamma(y_1, y_2|p,\alpha) Beta(p|m,n)dp$$
$$= \int_0^1 \left(\frac{\alpha}{1-p}\right)^{2\alpha} (y_1 y_2)^{\alpha-1} exp\left(-\frac{\alpha(y_1+y_2)}{1-p}\right)\frac{1}{\Gamma(\alpha)^2} p^{m-1}(1-p)^{n-1}\frac{\Gamma(m+n)}{\Gamma(m)\Gamma(n)}dp$$

This integral cannot be calculated analytically, so we approximate it numerically by quadrature. We calculate the likelihood for all 50 pairs of observations. We then maximize the likelihood product across the 50 parameter combinations, using the Nelder-Mead algorithm [104], i.e. to identify the combination of m, n and α parameters that give the highest likelihood. From the resulting m and n parameters we then calculate the mean of the proportion we seek to estimate and the 95% confidence intervals (the 2.5 and 97.5 percentiles of the beta distribution).

It is worth noting that the gamma distribution variance is $(1-p)^2/\alpha$, which increases as p reduces. This is consistent with our finding that the empirical estimates of the stochastic variance are negatively correlated with the p values (results not shown). The α parameter can thus be thought of as representing the inverse of the stochastic variance (precision) when the true proportion p is close to zero.

2.1 Additional complications when there is a non-zero probability that p = 0

In the preceding section we were implicitly assuming that p could not be zero. However, there may be some parameter combinations for which p is known to be zero. For example, if we are estimating the proportion of HIV infections that are attributable to binge drinking, but all of the assumed effects of binge drinking on sexual risk behaviour are zero, then the true PAF must also be zero. Suppose that a proportion z of our 50 parameter combinations have this property that they are known to be associated with a true proportion of zero. Then it would be more correct to describe the distribution of p values as being a mixture distribution with a probability mass z at zero and (with probability 1 – z) a beta distribution with parameters m and n.

The calculation of the likelihood is exactly the same as before for the parameter combinations that are known to be associated with non-zero values of p. But for the parameter combinations that are known to be associated with p = 0, we use the observed y_1 and y_2 values only for the purpose of estimating the stochastic variance, i.e. the likelihood is

$$L(y_1, y_2|\alpha) = \alpha^{2\alpha}(y_1y_2)^{\alpha-1}exp\big(-\alpha(y_1+y_2)\big)\frac{1}{\Gamma(\alpha)^2}.$$

The mean of the mixture distribution is then $(1-z)m/(m+n)$. If z is > 0.025, the lower limit of the 95% confidence interval will be 0. The upper limit of the 95% confidence interval will be $F^{-1}((0.975-z)/(1-z))$, where $F^{-1}(x)$ is the inverse of the cumulative beta distribution function evaluated at x.

3. Calibration to randomized controlled trial data

We adopt a semi-Bayesian approach to estimating the parameters in our model and quantifying the uncertainty around the model outputs. Prior distributions are specified to represent the uncertainty around (a) the effects of structural factors on HIV risk behaviour and (b) the effects of structural interventions. Likelihood distributions are calculated to represent the extent of the agreement between the model estimates and estimates from randomized controlled trials (RCTs) of the effectiveness of different structural interventions. The posterior distribution, which represents the set of estimates most consistent with both the prior distributions and the data that define the likelihood calculations, is difficult to estimate directly due to the long time required to run the model and the imprecision in the likelihood calculation, so we instead

identify a subset of the parameter space that yields the highest likelihood values. Each of these steps is described in more detail in the sections that follow.

3.1 Prior distributions

We distinguish here between the effects of structural factors on HIV risk behaviours and the effectiveness of structural interventions in changing structural factors. Examples of the former parameters include the effect of binge drinking on engaging in casual sex, and the effect of inequitable gender norms on condom use. As noted in the literature review, it is in many cases difficult to determine whether the observed associations between structural factors and HIV risk behaviours represent a 'true effect' or whether the observed associations are merely due to confounding. The prior distributions therefore need to reflect both (a) the uncertainty about whether there is a true effect, and (b) the uncertainty regarding the size of that effect, assuming it exists. To represent this uncertainty we use hurdle distributions. For example, a gamma hurdle distribution is used to represent the uncertainty around parameter A_1, the proportionate increase in men's rate of engaging in casual sex due to binge drinking. This means that

$$\Pr[A_1 = x] = \begin{cases} \theta & \text{if } x = 0 \\ \frac{(1-\theta)\lambda^{\alpha}x^{\alpha-1}}{\exp(\lambda x)\Gamma(\alpha)} & \text{if } x > 0 \end{cases}$$

where θ is the hurdle parameter, and α and λ are the shape and scale parameters of the gamma distribution respectively. The hurdle parameter θ therefore represents the prior belief in the probability of a null association. For the sake of setting assumed values of θ, we grade the strength of evidence for each of the hypothesized effect parameters into one of three categories:

I. Evidence from observational studies suggests a possible effect, but there are no RCTs confirming/suggesting that an effect exists.
II. There is some evidence from RCTs to suggest that an effect exists, but RCTs are not consistent or the evidence is considered weak.
III. There is evidence from at least two RCTs suggesting a significant effect, and the results from different trials are consistent.

We consider only RCTs conducted in sub-Saharan Africa in grading the evidence, as the social determinants of HIV risk behaviour are likely to be different outside of Africa, and we would therefore not expect structural interventions to have the same effects. The hurdle parameter θ is set to 0.75 for category I effects, 0.50 for category II effects and 0.25 for category III effects. For example, in the case of the effect of binge drinking on casual sex, there have been two RCTs that have assessed the effect of alcohol counselling interventions on levels of casual sex [75, 76], but only one found an effect of borderline significance [75] and in the other the reduction was non-significant [76]. We therefore grade the evidence as 'category II' and assign a 0.50 weight to the probability of no true effect.

We generally use gamma hurdle models for parameters that are defined on the range [0, ∞). However, many of the effect parameters are expressed as relative risks that are bounded on the range [0, 1]. In these case we use a beta hurdle model, with the hurdle at 1 rather than at 0, since a relative risk of 1 implies no effect. Mathematically, if β is the parameter for which a beta hurdle prior is specified,

$$\Pr[\beta = x] = \begin{cases} \theta & \text{if } x = 1 \\ \frac{(1-\theta)x^{m-1}(1-x)^{n-1}}{\mathrm{B}(m,n)} & \text{if } x < 1 \end{cases}$$

where θ is the hurdle parameter, and *m* and *n* are the parameters of the beta distribution.

Table S6 summarizes the parameters for which we have specified prior distributions in this analysis. These parameters have all been explained in previous sections (referenced in the final column of the table).

Table S6: Prior distributions for alcohol and gender norm parameters

Parameter	Prior type	Evidence grading	Mean*	Standard deviation*	Section
OR of condom use, per day of binge drinking, per week	Beta hurdle	II	0.65	0.20	1.6.3
Increase in casual sex in					
Male binge drinkers	Gamma hurdle	II	0.75	0.30	1.5.1
Female binge drinkers	Gamma hurdle	II	2.30	1.00	1.5.1
Probability of confounding between binge drinking and concurrency	Uniform	-	0.50	0.29	1.3.3
RR of binge drinking immediately after					
Single session of alcohol counselling	Beta	-	0.80	0.10	1.7
Multiple sessions of alcohol counselling	Beta	-	0.55	0.20	1.7
Annual probability of reverting to pre-intervention drinking pattern	Uniform	-	0.50	0.29	1.7
Effect of inequitable gender norms on					
Men’s entry into concurrent partnerships	Gamma hurdle	II	5.00	2.50	1.4
Men’s number of drinks per drinking day	Gamma hurdle	III	8.33	4.00	1.3.3
RR condom use in men endorsing inequitable gender norms	Beta hurdle	II	0.25	0.15	1.6.4
RR of entry into casual sex per 0.1 decrease in gender inequitable norm score	Beta hurdle	II	0.75	0.20	1.5.2
Odds of inequitable gender norms in low-risk men, compared to high-risk men	Beta	-	0.50	0.20	1.2
RR of men endorsing inequitable gender norms after gender-transformative interventions					
At individual level	Uniform	-	0.50	0.29	1.8
At community level	Uniform	-	0.50	0.29	1.8
Annual probability of reverting to pre-intervention gender norms	Uniform	-	0.50	0.29	1.8

OR = odds ratio. RR = relative risk. * In the case of gamma hurdle and beta hurdle distributions, the mean and standard deviation are for the gamma and beta components of the distribution respectively (i.e. ignoring the probability of a null association).

3.2 Likelihood function

We calibrated the model to data from randomized controlled trials of alcohol counselling and gender-transformative interventions in sub-Saharan Africa. Relevant trials were identified from systematic reviews of alcohol counselling interventions in low- and middle-income countries [106] and in HIV-positive adults [107], and from systematic reviews of gender-transformative interventions [100-103].

In the case of alcohol counselling interventions, we included only those studies that targeted individuals who were judged to be hazardous drinkers at baseline (in many of the included studies, this was defined as a baseline AUDIT score of 8 or higher). Some studies were

excluded because the control group received an intervention that was considered likely to reduce risky drinking [91]. Some studies were excluded because they targeted counselling at a group level, and therefore were not limited to hazardous drinkers [108-110]. Studies were also excluded if they involved pharmacological treatments or opinion leader interventions [111]. We distinguish between single-session interventions and multi-session interventions, as previous reviews suggest the latter are likely to be more effective [94].

In the case of gender-transformative interventions, we included only those trials in which the intervention involved critical reflection of gender norms and gender inequalities. We excluded those studies that focused exclusively on the prevention of intimate partner violence without addressing broader gender norms [112, 113] and trials that were non-randomized [112]. Because we were particularly interested in the ways in which inequitable gender norms shape male risk behaviours, we consider only outcomes reported for men. Trials that did not report results disaggregated by sex [114], or that recruited only women, were therefore excluded.

For each of the included trials, we recorded the type of intervention, and for each outcome that was reported in the trial we recorded

- The type of outcome (e.g. AUDIT score, condom use at last sex, HIV incidence)
- The demographic characteristics (sex and age range, and whether participants were in school or not)
- The type of statistical measure that was used to compare the intervention and control arms (e.g. log odds ratio, relative risk, or absolute change)
- The difference in outcome between intervention and control arms, and the standard error associated with the difference
- The follow-up duration to which the outcome measure related (in months)

We limited our analysis to outcomes that could reasonably be matched to outcomes simulated in our model. These outcomes included:

- Proportion of days on which alcohol is consumed
- Number of drinks per drinking day
- Any binge drinking in the last month
- AUDIT score
- AUDIT-C score
- Alcohol abstinence in the last month
- Probability of endorsing inequitable gender norms
- Recent unprotected sex
- Condom use at last sex
- Multiple current partnerships (concurrency)
- Multiple partners in the last year
- Casual or transactional sex in the last year
- Currently engaging in casual or transactional sex
- Cumulative HIV incidence
- Cumulative HSV-2 incidence
- Cumulative incidence of curable STIs (gonorrhoea, chlamydia or trichomoniasis)

In some cases we included estimates of intervention effects even if the recorded outcome was not exactly consistent with the model definition, but the definitions were deemed close enough. For example, in the SASA! trial, one of the outcomes was any concurrent partnerships in the last year [115], which we compared to the model estimate of multiple current partnerships, on the assumption that the proportional change in the outcome due to the intervention would be

much the same whether the outcome was defined at a point in time or cumulatively over one year. Although our model does not include ‘transactional sex’ per se, as noted in section 1.5, we expect that casual sex relationships would tend to be more transactional in nature [64, 65], and we therefore compare our modelled casual sex outcomes against transactional sex outcomes in trials.

It is also worth noting that in a few cases (specifically cumulative HSV-2 incidence and cumulative HIV incidence) we replaced the actual trial duration with a longer trial duration when simulating the modelled trial outcome, because HSV-2 and HIV incidence are rare outcomes, and using a longer simulated trial duration therefore helps to reduce the stochastic variation in the model outputs.

Table S7 summarizes the data recorded for each of the included trials. In total 9 RCTs of alcohol counselling interventions and 4 RCTs of gender-transformative interventions were included (the Abramsky and Kyegombe studies related to the same RCT). A total of 47 outcomes were recorded in these 13 trials (27 from the alcohol counselling trials and 20 from the gender-transformative intervention trials).

Table S7: Randomized controlled trial data included in model calibration

Intervention	Study	Outcome	Population	Timing (months)	Measure	Effect	SE
Alcohol counselling: Single session	Kalichman et al [75]	Unprotected sex with recent partners	M+F, 18+	3	log(OR)	-1.099	0.561
		Unprotected sex with recent partners	M+F, 18+	6	log(OR)	-0.262	0.561
		Current casual sex	M+F, 18+	3	log(OR)	-0.916	0.467
		Current casual sex	M+F, 18+	6	log(OR)	-0.693	0.467
	Kalichman et al [90]	Condom use at last sex	M+F, 18+	3	log(OR)	2.351	1.103
		Condom use at last sex	M+F, 18+	6	log(OR)	1.668	0.859
	Huis in 't Veld et al [38]	AUDIT score	M+F, 18+	5	Absolute	-0.718	0.947
		AUDIT score	M+F, 18+	12	Absolute	-1.163	0.946
	Wandera et al [37]	AUDIT-C score	M+F, 18+	6	Absolute	-0.070	0.321
	Zule et al [116]	Abstinence in last month	F, 18-33	12	log(OR)	1.280	0.575
Alcohol counselling: Multiple sessions	Papas et al [95]	% of drinking days	M+F, 18+	1	Absolute	-0.250	0.064
		% of drinking days	M+F, 18+	2	Absolute	-0.220	0.068
		% of drinking days	M+F, 18+	3	Absolute	-0.170	0.070
		# drinks per drinking day	M+F, 18+	1	Absolute	-2.900	0.931
		# drinks per drinking day	M+F, 18+	2	Absolute	-3.500	1.089
		# drinks per drinking day	M+F, 18+	3	Absolute	-2.500	1.105
	L'Engle et al [92]	Binge drinking in the last month	F, 18+	6	log(RR)	-0.942	0.358
		Binge drinking in the last month	F, 18+	12	log(RR)	-0.918	0.393
		Cumulative NG/CT/TV incidence	F, 18+	6	log(OR)	0.166	0.221
		Cumulative NG/CT/TV incidence	F, 18+	12	log(OR)	-0.274	0.282
		Unprotected sex with recent partners	F, 18+	6	log(OR)	0.128	0.146
		Unprotected sex with recent partners	F, 18+	12	log(OR)	0.030	0.147
	Wechsberg et al [76]	Current casual sex	F, 18+	6	log(OR)	-0.010	0.156
		Current casual sex	F, 18+	12	log(OR)	-0.171	0.167
		Unprotected sex with recent partners	F, 18+	6	log(OR)	0.010	0.156
		Unprotected sex with recent partners	F, 18+	12	log(OR)	-0.140	0.255
	Madhombiro et al [36]	AUDIT score	M+F, 18+	6	Absolute	-3.090	0.840

Gender-transformative interventions: Community-based	Pettifor et al [29]	Endorsing inequitable gender norms	M, 18-35	24	log(OR)	-0.329	0.134
		Cumulative partners (last year)	M, 18-35	24	log(OR)	-0.315	0.421
		Binge drinking in the last month	M, 18-35	24	log(OR)	0.425	0.645
		Condom use at last sex	M, 18-35	24	log(OR)	0.300	0.218
	Abramsky et al [115]	Concurrency	M, 18-49	48	log(RR)	-0.562	0.237
	Kyegombe et al [93]	Condom use at last sex	M, 18-49	48	log(RR)	0.708	0.261
Gender-transformative interventions: Individual-based	Gibbs et al [30]	Endorsing inequitable gender norms	M, 18-30	12	log(OR)	-0.423	0.103
		Endorsing inequitable gender norms	M, 18-30	24	log(OR)	-0.024	0.055
		AUDIT score	M, 18-30	12	Absolute	-2.090	0.987
		AUDIT score	M, 18-30	24	Absolute	-1.100	0.617
		Cumulative casual sex	M, 18-30	12	log(OR)	-0.528	0.280
		Cumulative casual sex	M, 18-30	24	log(OR)	-0.030	0.220
	Jewkes et al [32]	Cumulative casual sex	M, 15-26	12	log(OR)	-0.236	0.147
		Cumulative casual sex	M, 15-26	24	log(OR)	-0.163	0.158
		Binge drinking in the last month	M, 15-26	12	log(OR)	-0.386	0.166
		Binge drinking in the last month	M, 15-26	24	log(OR)	0.095	0.155
		Condom use at last sex	M, 15-26	12	log(OR)	0.231	0.163
		Condom use at last sex	M, 15-26	24	log(OR)	-0.128	0.162
		Cumulative HSV-2 incidence	M, 15-26	24*	log(RR)	-0.335	0.353
		Cumulative HIV incidence	M, 15-26	24*	log(RR)	0.082	0.376

CT = *Chlamydia trachomatis* (chlamydia), F = female, M = male, NG = *Neisseria gonorrhoeae* (gonorrhoea), OR = odds ratio, RR = risk ratio, SE = standard error, TV = *Trichomonas vaginalis* (trichomoniasis). * For the purpose of simulating the trial outcome, we used a longer trial duration (84 months) in order to reduce stochastic variation.

For each trial, and for each outcome measured in that trial, we calculate a likelihood that measures the extent of consistency between the outcome measured in the trial and the outcome predicted by the model. The outcome predicted by the model is calculated by simulating the outcome in the intervention arm (starting in 2005, we assign individuals to receive the intervention if they are eligible, and track their outcomes over the term of the trial) and comparing this to the simulated outcome in the control arm (restarting the model from 2005, in the same simulated individuals who would have qualified for the intervention, but now assuming that they do not receive the intervention). The likelihood is calculated on the assumption that the observed trial outcome is normally distributed, with mean equal to the model prediction and variance calculated from the standard error reported for the trial outcome.

A limitation of this approach to defining the likelihood is that it implicitly assumes a 'fixed effects' model, i.e. we are not accounting for the possibility that factors other than random error could account for differences between the modelled trial outcome and the observed outcome. However, given that the confidence intervals around the trial outcomes are generally quite wide, we do not expect that using a random effects model would substantially change the outcomes. Another limitation is that we have somewhat arbitrarily set the intervention start year in the model to 2005, and have not tried to match this to the actual start years in the trial. However, we do not anticipate that there would be major changes in intervention effectiveness over time, and using the same start year for all model simulations has the advantage of reducing the number of model runs that are required.

3.3 Selecting the best-fitting parameter combinations

We draw an initial sample of 5000 parameter combinations from the prior distributions described in section 3.1, and for each parameter combination we run the model twice (with different seeds in the random number generator), calculate the likelihood described in section 3.2 for each model run, and then calculate the geometric average of the two likelihood values for each parameter combination. We then select the 50 parameter combinations that have the highest average likelihood values. The reason for running the model twice for each parameter combination is to reduce the influence of stochastic variation in the likelihood calculation. However, likelihood values were found to be highly correlated across the two sets of results, and the stochastic variation is therefore unlikely to have much effect on the selection of the 50 'best fitting' parameter combinations.

3.4 Best-fitting parameter combinations

Table S8 compares the best-fitting parameter combinations with the prior distributions specified in Table S6. The table shows both the best-fitting parameter values for the overall likelihood and the best-fitting parameters for the different intervention types, i.e. considering the likelihood for each intervention type separately. (In some cases the 'best-fitting' parameter for a particular intervention type is not shown because the parameter does not influence any of the intervention outcomes – for example, we would not expect the efficacy of single-session alcohol counselling in reducing alcohol consumption to influence the outcomes for any of the other interventions.)

Table S8: Best-fitting parameter combinations

Parameter	Prior mean (95% CI)	Alcohol counselling interventions: Single-session	Multi-session	Combined	Gender-transformative interventions: Community-level	Individual-level	Combined	All
OR of condom use, per day of binge drinking, per week	0.83 (0.29-1.00)	**0.39 (0.15-0.70)**	**0.97 (0.78-1.00)**	**0.93 (0.62-1.00)**	0.77 (0.28-1.00)	0.80 (0.22-1.00)	0.81 (0.36-1.00)	0.84 (0.44-1.00)
Increase in casual sex in								
Male binge drinkers	1.38 (1.00-2.30)	1.46 (1.00-2.27)	1.34 (1.00-2.25)	1.39 (1.00-2.27)	1.45 (1.00-2.33)	1.33 (1.00-2.14)	1.28 (1.00-2.30)	**1.52 (1.00-2.25)**
Female binge drinkers	2.15 (1.00-5.15)	**2.65 (1.00-5.58)**	**1.35 (1.00-2.87)**	**1.46 (1.00-3.28)**	1.93 (1.00-5.99)	2.30 (1.00-4.30)	2.24 (1.00-4.66)	**1.55 (1.00-4.19)**
Probability of confounding between binge drinking and concurrency	0.50 (0.03-0.98)	0.47 (0.04-0.98)	0.52 (0.02-0.99)	0.55 (0.04-0.98)	0.55 (0.05-0.98)	**0.33 (0.00-0.94)**	0.44 (0.01-0.97)	0.43 (0.02-0.94)
RR of binge drinking immediately after								
Single session of alcohol counselling	0.80 (0.57-0.95)	**0.83 (0.59-0.90)**	-	**0.86 (0.77-0.95)**	-	-	-	0.84 (0.76-0.94)
Multiple sessions of alcohol counselling	0.55 (0.16-0.90)	-	**0.40 (0.35-0.48)**	**0.41 (0.35-0.49)**	-	-	-	**0.42 (0.31-0.54)**
Annual probability of reverting to pre-intervention drinking pattern	0.50 (0.03-0.98)	**0.32 (0.01-0.97)**	**0.20 (0.03-0.42)**	**0.19 (0.01-0.41)**	-	-	-	**0.32 (0.02-0.68)**
Effect of inequitable gender norms on								
Men's entry into concurrent partnerships	2.50 (0.00-9.7)	-	-	-	**5.01 (0.00-12.9)**	3.30 (0.00-13.2)	3.18 (0.00-9.70)	3.21 (0.00-8.72)
Men's number of drinks per drinking day	6.25 (0.00-16.7)	-	-	-	5.79 (0.00-14.3)	**10.8 (0.80-26.5)**	**9.91 (0.00-26.5)**	7.32 (0.00-21.3)
RR condom use in men endorsing inequitable gender norms	0.63 (0.05-1.00)	-	-	-	**0.21 (0.03-0.91)**	0.56 (0.10-1.00)	0.56 (0.05-1.00)	0.60 (0.04-1.00)
RR of entry into casual sex per 0.1 decrease in gender inequitable norm score	0.88 (0.35-1.00)	-	-	-	0.89 (0.36-1.00)	**0.85 (0.68-1.00)**	**0.81 (0.47-1.00)**	**0.86 (0.60-1.00)**
Odds of inequitable gender norms in low-risk men, compared to high-risk men	0.50 (0.13-0.87)	-	-	-	0.49 (0.14-0.87)	0.50 (0.11-0.82)	0.51 (0.19-0.82)	0.53 (0.17-0.79)
RR of men endorsing inequitable gender norms after gender-transformative interventions								
At individual level	0.50 (0.03-0.98)	-	-	-	-	**0.23 (0.01-0.51)**	**0.34 (0.02-0.71)**	**0.36 (0.01-0.77)**
At community level	0.50 (0.03-0.98)	-	-	-	0.45 (0.02-0.75)	-	**0.31 (0.01-0.83)**	0.45 (0.03-0.98)
Annual probability of reverting to pre-intervention gender norms	0.50 (0.03-0.98)	-	-	-	**0.32 (0.03-0.56)**	**0.74 (0.63-0.84)**	**0.67 (0.46-0.81)**	**0.67 (0.47-0.85)**

OR = odds ratio. RR = relative risk. Where the mean of the best-fitting parameters differs significantly (at 5% level) from the prior mean, this is indicated in bold.

The table also indicates (in bold) which of the means of the best-fitting parameter parameters differ significantly from the prior mean. In the case of the alcohol counselling interventions, the best-fitting parameters suggest a greater efficacy of multi-session interventions than assumed *a priori*, but a slightly poorer efficacy of single-session interventions than assumed *a priori*. In both cases, the rate of reverting to pre-intervention drinking patterns is significantly lower than the prior mean. The data from multi-session and single-session counselling interventions are contradictory regarding the likely effect of binge drinking on condom use: the former suggest a very small effect, while the latter suggest a greater effect than assumed *a priori*.

The data from gender-transformative intervention trials suggest greater effects of inequitable gender norms on men's alcohol consumption and condom use than assumed *a priori*. Although individual-level gender-transformative interventions appear more effective than community-level interventions in the short term, their effect appears significantly less durable over the longer term (i.e. men receiving individual-level interventions appear more likely to revert to their baseline risk behaviours who are exposed to community-level gender-transformative interventions).

3.5 Calibration outputs

Figure S6 shows the model calibration to the structural driver outcome data. Most of the model estimates of the impact of alcohol counselling and gender-transformative interventions on levels of alcohol and inequitable gender norms fall well within the 95% confidence intervals around the corresponding trial outcomes. However, the modelled impact of individual-level gender-transformative interventions on alcohol outcomes is more modest than observed in the RCTs of Jewkes *et al* [32] and Gibbs *et al* [30]. This suggests that the model may be under-estimating the effect of inequitable gender norms on alcohol levels in men, or else that the gender-transformative interventions influence alcohol consumption through other mechanisms that are not mediated by gender norms.

Figure S7 shows the model calibration to the risk behaviour outcome data. Again, most of the model estimates of the impact of alcohol counselling and gender-transformative interventions on sexual risk behaviour fall within the 95% confidence intervals around the corresponding trial outcomes. However, the two trials conducted by Kalichman *et al* [75, 90] report substantially greater reductions in unprotected sex and casual/transactional sex than predicted by our model. This may be because the interventions tested in these two trials were not purely alcohol counselling interventions – they also included significant HIV risk reduction counselling. The model is also not very consistent with the substantial changes in condom use and concurrency in the 'SASA!' trial, as reported by Kyegombe *et al* [93] and Abramsky *et al* [115] respectively. It has been argued that there is a risk of bias in this trial because of the political disturbances in Uganda at the time of the trial [101], although it is not clear how such disturbances would create a bias towards an exaggerated intervention effect.

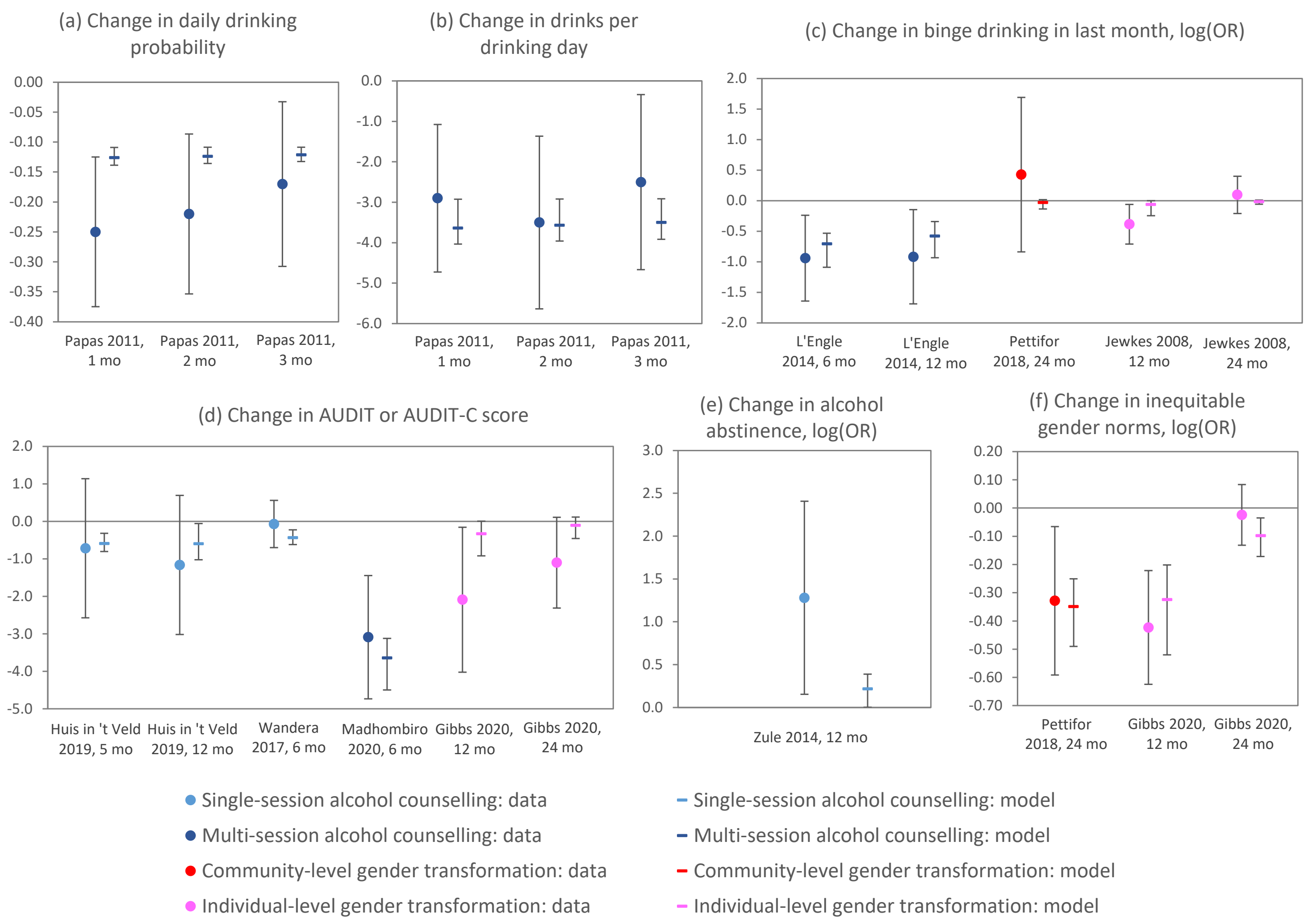

Figure S6: Calibration to alcohol and gender norm outcome data

In all panels the observed trial outcomes (dots) are compared against the model predictions of the trial impact (horizontal dashes), averaging the 50 best-fitting model results. Vertical lines represent 95% confidence intervals. In panels (c), (e) and (f), the measure of intervention impact is the logarithm of the odds ratio (comparing the intervention arm to the control arm), and in the other panels the measure of intervention impact is the absolute change in the outcome (comparing the intervention arm to the control arm). In panel (d) the AUDIT and AUDIT-C scores are grouped together, although they are calculated differently in the model.

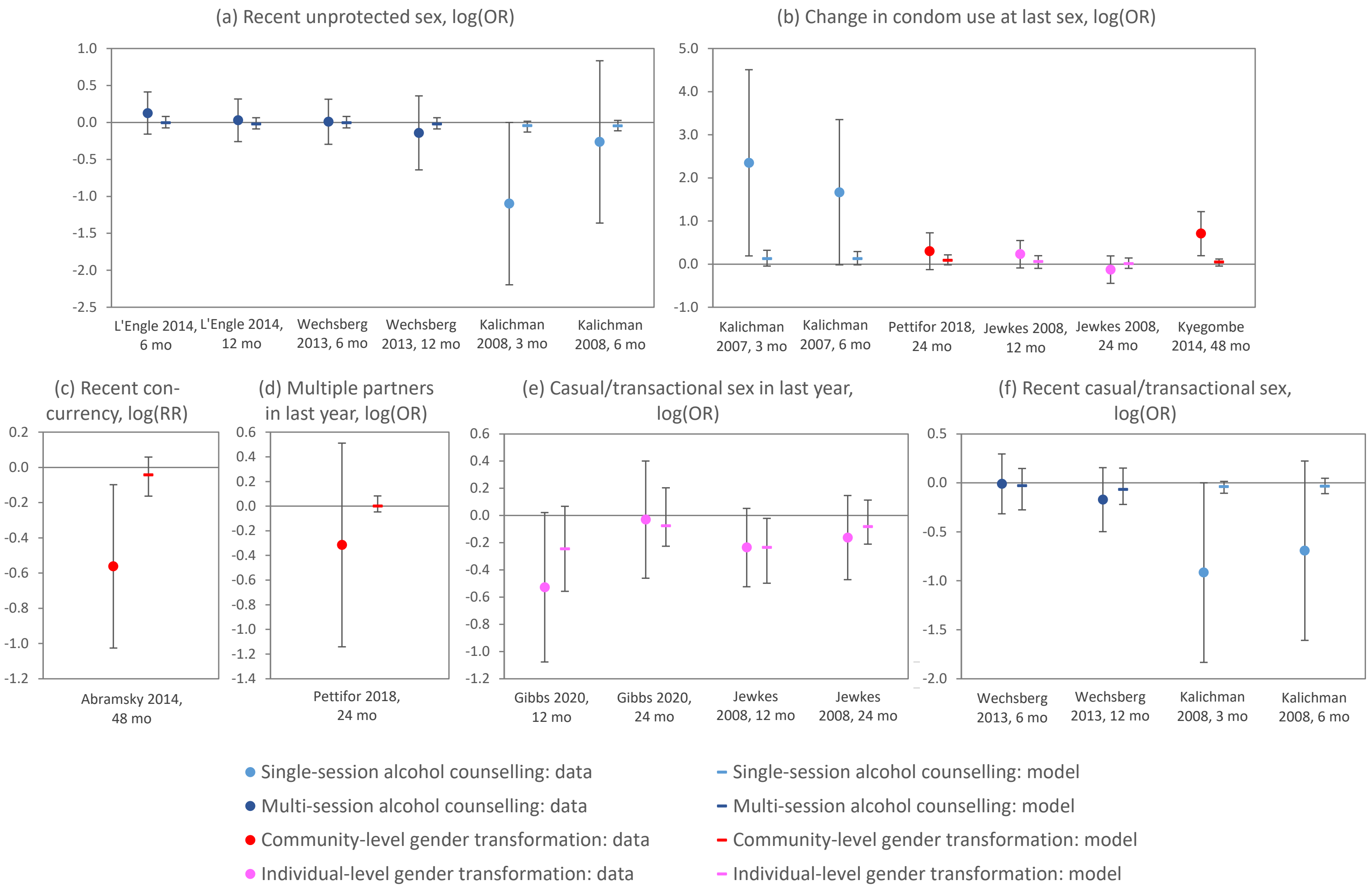

Figure S7: Calibration to sexual risk behaviour outcome data

In all panels the observed trial outcomes (dots) are compared against the model predictions of the trial impact (horizontal dashes), averaging the 50 best-fitting model results. Vertical lines represent 95% confidence intervals. In all panels, the measure of intervention impact is the logarithm of the odds ratio (OR) or relative risk (RR) when comparing the intervention arm to the control arm.

Figure S8 shows the model calibration to the health outcome data (limited to HIV and STI incidence). There are relatively few RCTs that included biological endpoints, and the confidence intervals around the RCT effects on these outcomes are generally wide. The best-fitting model results suggest that gender-transformative and alcohol counselling interventions have negligible impact on HIV and STI incidence in the short term.

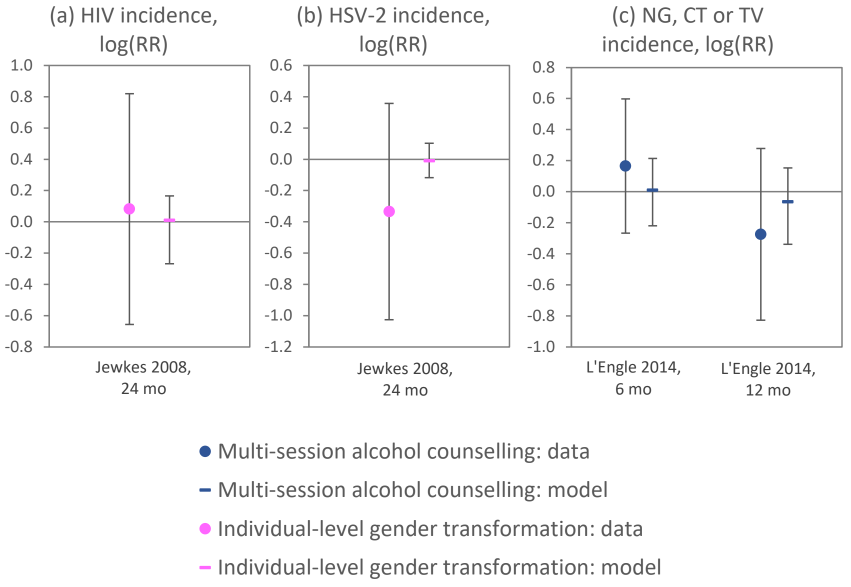

Figure S8: Calibration to biological outcome data

In all panels the observed trial outcomes (dots) are compared against the model predictions of the trial impact (horizontal dashes), averaging the 50 best-fitting model results. Vertical lines represent 95% confidence intervals. In all panels, the measure of intervention impact is the logarithm of the relative risk (RR) when comparing the intervention arm to the control arm. CT = *Chlamydia trachomatis* (chlamydia), HSV-2 = herpes simplex virus type 2 (genital herpes), NG = *Neisseria gonorrhoeae* (gonorrhoea), TV = *Trichomonas vaginalis* (trichomoniasis).

4. Additional results

4.1 Validation against household survey data

We compared the model estimates of the association between inequitable gender norms, binge drinking and sexual risk behaviour, with the associations measured in four national household surveys. The four surveys are the Human Science Research Council (HSRC) surveys of 2008 [117], 2012 [118] and 2017 [119], and the 2016 Demographic and Health Survey [56]. (Information on HIV risk behaviours was lacking for the 2005 HSRC survey, and this survey has therefore not been included.) Because gender norms, risk behaviours and alcohol consumption patterns vary between race groups in South Africa, and we wanted to avoid potential confounding due to possibly mis-specified race effects, we have limited this comparison to black South Africans (who account for approximately 80% of the South African population). We have also limited the comparison to sexually active adults (ages 15-49). All comparisons are shown separately for men and women (although in the case of inequitable gender norms, we only show results for men, since we do not model inequitable gender norms in women). Because none of the surveys directly measured inequitable gender norms, we use acceptance of wife beating as a survey proxy for inequitable gender norms (the question was

not included in the 2008 HSRC survey); in the model we arbitrarily classify men as endorsing inequitable gender norms if their inequitable norms score is 0.4 or greater (i.e. roughly two times the population mean). ‘Binge drinking’ was defined as consuming at least 5 drinks on a single day within the last month, ‘condom use’ was defined as condom use at last sex, and ‘multiple partners’ was defined as having two or more sexual partners in the last 12 months. The measure of association used in these comparisons is the log of the odds ratio, and for each of the surveys the log odds ratio is calculated from the individual-level survey data.

Figure S9 shows the results of the model validations. For the most part, the model results are consistent with the associations measured in the household surveys, although the confidence intervals around the model estimates are quite wide, reflecting the substantial uncertainty that remains in the relationship between alcohol, inequitable gender norms and sexual risk behaviour, even after the model has been calibrated to the RCT data. The modelled associations between binge drinking and multiple partners in women appear too low, relative to the associations measured in the survey. This suggests that there may be sources of association that we have not accounted for in our model (for example, religiosity [52]), despite our attempts to make some allowance for this association (as described in the final paragraph of section 1.3.3).

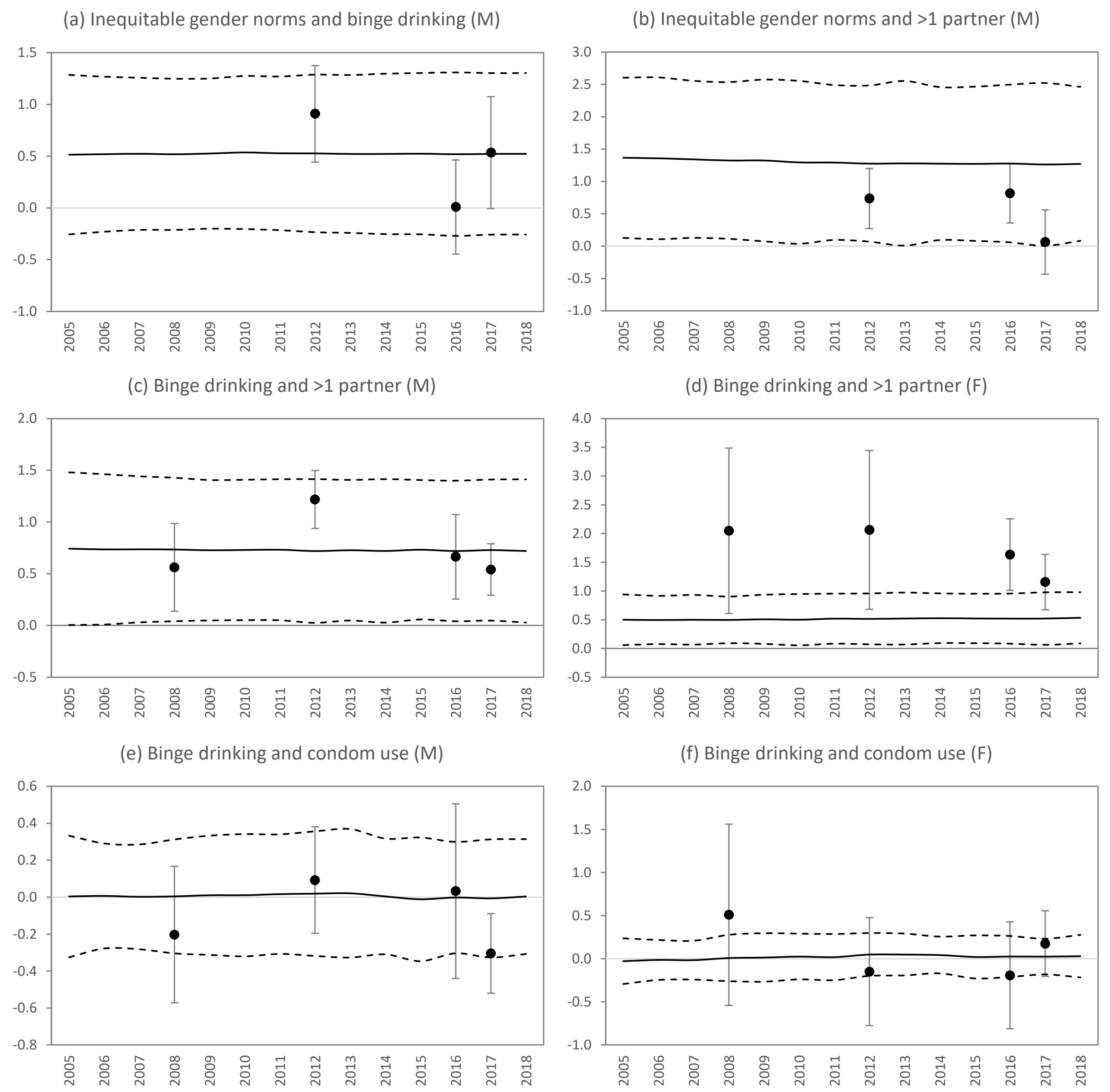

Figure S9: Modelled associations (on log(OR) scale) between binge drinking, inequitable gender norms and sexual risk behaviours, compared against associations measured in national surveys

Dots represent data for the black South African population (aged 15-49), and error bars reflect 95% confidence intervals. Solid lines represent the average of the modelled associations (across the 50 best-fitting parameter combination) and dashed lines reflect the 95% confidence intervals around the modelled associations. F = females, M = males.

4.2 Calibration to HIV data

A number of changes were made to the model described previously [1], in order to maintain consistency with the HIV prevalence targets that we have previously used in model calibration. Briefly:

- The ‘base’ rates of short-term partnership formation (the c_g parameters in section 4.3 of the previous report [1]) have been reduced to 2 and 4 per annum, in males and females respectively (from the previous values of 7.3 and 14.6). This is to compensate for the

introduction of casual sex into MicroCOSM, which substantially increases the density of the simulated sexual networks.

- The ultimate odds of condom use, relative to the odds of condom use in 1998 (the $\exp(\kappa^2)$ parameter in section 4.9.1 of the previous report [1]), has been reduced, both in the case of short-term partnerships, and in the case of sex worker-client interactions (Table S9). This is to compensate for the adjustments to the condom model described in section 1.6 of the supplementary materials.

Table S9: Changes in condom parameters

Parameter	Symbol	New	Previous
Ultimate odds of condom use, relative to 1998	$\exp(\kappa^2)$		
Short-term relationships		5.56	6.4
Sex worker-client relationships		6.29	7.2
Shape parameter controlling speed of behaviour change	Q		
Short-term relationships		3.05	2.8
Sex worker-client relationships		4.0	4.0

- The standard deviation representing inter-individual variation in condom preferences (the Ω_i values on p. 93 of the previous report [1]) was reduced to 1.0 (from the previous value of 1.4). This is because some of the inter-individual variation in condom preferences is due to differences in alcohol consumption and gender norms – variables that were not included in our previous model.
- The HIV transmission probabilities per act of unprotected sex, in long-term relationships, sex worker-client relationships and MSM relationships, were fixed at the medians of the 100 best-fitting parameters that were identified when the model was previously calibrated to South African HIV data (Table 9.1 of the previous report [1]). However, we assumed slightly lower transmission probabilities (relative to the best-fitting parameters identified previously) in the case of short-term relationships: 0.0017 in the case of male-to-female transmission and 0.00075 in the case of female-to-male transmission. This change was made in order to improve the calibration to the HIV prevalence data, and also to achieve greater consistency between the per-act transmission probabilities in short-term and long-term relationships.
- The initial HIV prevalence parameter and the parameters determining relative HIV infectivity by disease stage (which were varied in the previous model calibration) were fixed at the prior means rather than at the median of the best-fitting parameters found previously (see Table 8.1 of the previous report [1]). This was because the prior means were found to be more consistent with the HIV prevalence data in the updated model presented here.
- The HIV mortality parameters for ART patients (the k_g and M_g parameters in section 6.3 of the previous report [1]) were reduced by 50%. Although the main reason for this change was to bring estimates of AIDS mortality in recent years more in line with the Thembisa model [121], this change was also prompted by concern that previous estimates of 'excess deaths' in ART patients (which were the basis for our previous assumptions about HIV mortality in ART patients [122]) might be a poor indication of HIV-specific mortality.
- We reduced the AIDS mortality rate in ART-naïve individuals by the proportion $\Psi\Lambda(t)^{1-v}h(t)$, where Ψ is the probability that HIV-related death is immediately preceded by an opportunistic infection (OI), $\Lambda(t)$ is the proportion of OI patients tested for HIV in year t, v is the individual's diagnosis history (1 if previously diagnosed, 0 if not), and $h(t)$ is the probability that an HIV-diagnosed OI patient starts ART, in year

t. The rates of HIV testing in OI patients ($\Lambda(t)$) and rates of linkage to ART in OI patients ($h(t)$) are the same as assumed in the previous version of MicroCOSM (see Tables 5.2.1 and 5.3.2 respectively in the previous report [1]). The probability of an OI immediately preceding ART has been set to 0.75, based on a South African study of causes of death in people living with HIV (mostly untreated) [123]. These adjustments were made in order to bring the model estimates of AIDS deaths in the early ART period more in line with the Thembisa estimates [121]. (Although the previous MicroCOSM model did allow for HIV testing in OI patients and subsequent ART initiation, the modelling of HIV mortality was independent of the modelling of OIs, and hence the model did not capture the targeting of ART to the more clinically severe HIV cases, in the early stages of the ART rollout.)

- We also changed the model of ART initiation so that the rate of ART initiation, conditional on ART eligibility and receipt of an HIV diagnosis, is a continuous function of the CD4 count, rather than a 'step function'. If $A(t)$ is the 'base' ART initiation rate in year t (specified previously for individuals with CD4 counts of <200 cells/μl), then the rate of ART initiation in individuals with a CD4 count of x is set to $A(t)\chi^{(x-100)/100}$, where χ is the factor by which the rate of ART initiation reduces, per 100-cell increase in the CD4 count. We set χ to 0.8, based on a South African study of ART initiation rates [124].
- Rates of ART initiation in HIV-diagnosed individuals were updated to be consistent with Thembisa version 4.5 [125].

Figure S10 shows that after making these changes to the model, we obtain acceptable calibration of the model to HIV prevalence estimates from nationally-representative household surveys. The model results suggest some degree of 'levelling off' in male HIV prevalence, while HIV prevalence in females has been steadily increasing.

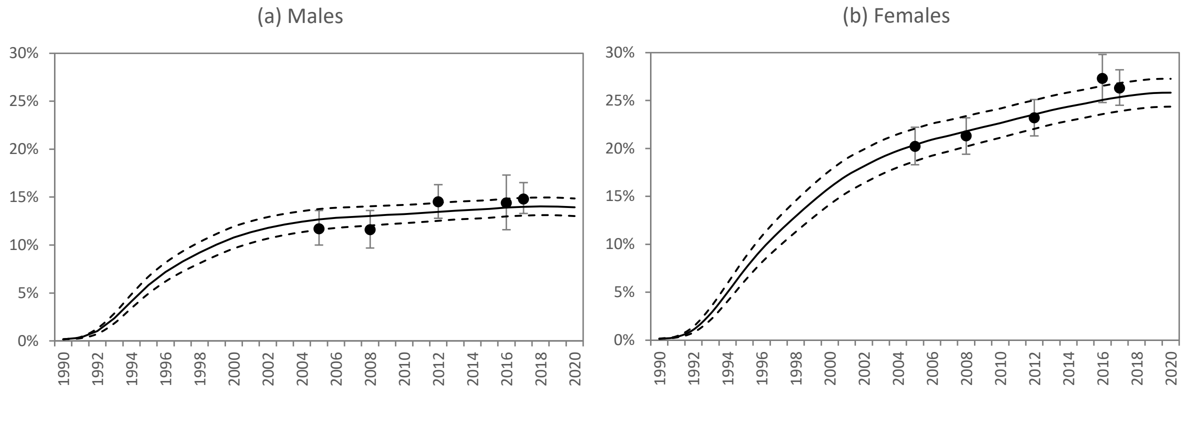

Figure S10: HIV prevalence in adults aged 15-49

The solid line represents the average results from the 50 model simulations that yield the best fit to the structural RCT data, and the dashed lines represent the confidence intervals, calculated from the standard errors around the means. Dots represent the HIV prevalence estimates from HSRC household surveys [119] and the 2016 DHS [56], and vertical error bars represent 95% confidence intervals around these survey estimates.

Figure S11 compares the updated model estimates of annual AIDS deaths with the estimates from the most recent Thembisa model, which is calibrated to South African vital registration data [125]. Although MicroCOSM slightly under-estimates levels of AIDS mortality in the 1990s, when compared with Thembisa, it slightly over-estimates levels of AIDS mortality in the 2000s.

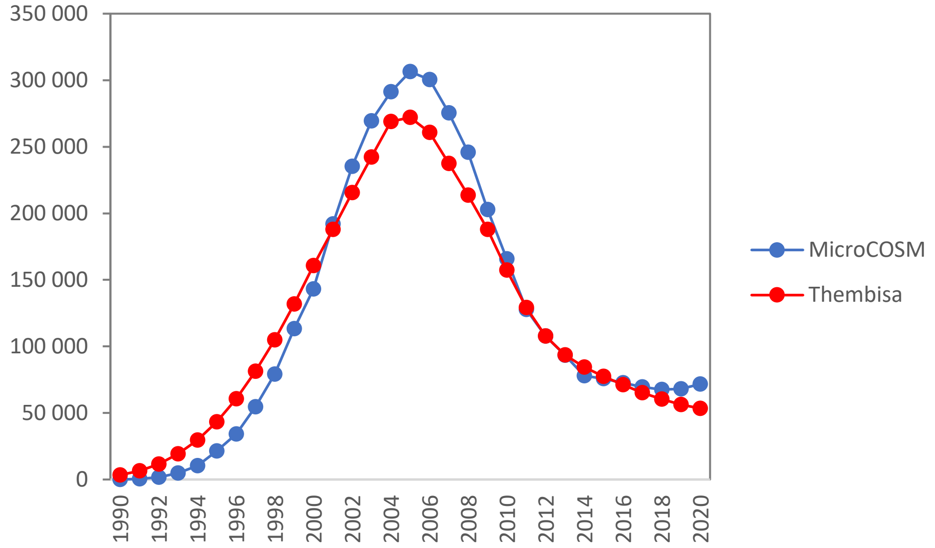

Figure S11: Annual AIDS deaths

MicroCOSM estimates are calculated based on the average results from the 50 model simulations that yield the best fit to the structural RCT data. Thembisa estimates are the posterior means obtained from version 4.5 of the model [125].

References

1. Johnson LF, Kubjane M, Moolla H. MicroCOSM: a model of social and structural drivers of HIV and interventions to reduce HIV incidence in high-risk populations in South Africa. *BioRxiv* 2018:310763.
2. McCrae RR, Stone SV. Personality. In: *Cambridge Handbook of Psychology, Health and Medicine*. Edited by Baum A, Newman S, Weinman J, West R, McManus C. Cambridge: Cambridge University Press; 1997. pp. 29-34.
3. Goodwin RD, Friedman HS. Health status and the five-factor personality traits in a nationally representative sample. *Journal of Health Psychology* 2006; **11**:643-654.
4. Bogg T, Roberts BW. Conscientiousness and health-related behaviors: a meta-analysis of the leading behavioral contributors to mortality. *Psychological Bulletin* 2004; **130**:887-919.
5. Hakulinen C, Elovainio M, Batty GD, Virtanen M, Kivimaki M, Jokela M. Personality and alcohol consumption: Pooled analysis of 72,949 adults from eight cohort studies. *Drug Alcohol Depend* 2015; **151**:110-114.
6. Adan A, Forero DA, Navarro JF. Personality traits related to binge drinking: a systematic review. *Frontiers in Psychiatry* 2017; **8**:134.
7. Schmitt DP. The big five related to risky sexual behaviour across 10 world regions: differential personality associations of sexual promiscuity and relationship infidelity. *European Journal of Personality* 2004; **18**:301-319.
8. Allen MS, Walter EE. Linking big five personality traits to sexuality and sexual health: A meta-analytic review. *Psychological Bulletin* 2018; **144**:1081-1110.
9. Poropat AE. A meta-analysis of the five-factor model of personality and academic performance. *Psychological Bulletin* 2009; **135**:322-338.
10. Roberts BW, Kuncel NR, Shiner R, Caspi A, Goldberg LR. The power of personality: the comparative validity of personality traits, socioeconomic status, and cognitive ability for predicting important life outcomes. *Perspectives on Psychological Science* 2007; **2**:313-345.
11. Nel JA, Valchev VH, Rothmann S, van de Vijver FJR, Meiring D, de Bruin GP. Exploring the personality structure in the 11 languages of South Africa. *Journal of Personality* 2012; **80**:915-948.
12. Fetvadjiev VH, Meiring D, van de Vijver FJ, Nel JA, Hill C. The South African Personality Inventory (SAPI): a culture-informed instrument for the country's main ethnocultural groups. *Psychological Assessment* 2015; **27**:827-837.
13. van Aarde N, Meiring D, Wiernik BM. The validity of the Big Five personality traits for job performance: Meta-analyses of South African studies. *International Journal of Selection and Assessment* 2017; **25**:223-239.
14. McCrae RR, Terracciano A. Personality profiles of cultures: aggregate personality traits. *Journal of Personality and Social Psychology* 2005; **89**:407-425.
15. Costa PT, McCrae RR. Longitudinal stability of adult personality. In: *Handbook of Personality Psychology*. Edited by Hogan R, Johnson J, Briggs S. San Diego, USA: Academic Press; 1997. pp. 269-290.
16. Roberts BW, Walton KE, Viechtbauer W. Patterns of mean-level change in personality traits across the life course: a meta-analysis of longitudinal studies. *Psychological Bulletin* 2006; **132**:1-25.
17. Lam D, Ardington C, Leibbrandt M. Schooling as a lottery: Racial differences in school advancement in urban South Africa. *J Dev Econ* 2011; **95**:121-136.

18. Migali G, Zucchelli E. Personality traits, foregone healthcare and high school dropout: Evidence from US adolescents. *Journal of Economic Psychology* 2017; **62**:98-119.
19. Gottert A, Barrington C, McNaughton-Reyes HL, Maman S, MacPhail C, Lippman SA, *et al.* Gender norms, gender role conflict/stress and HIV risk behaviors among men in Mpumalanga, South Africa. *AIDS Behav* 2018; **22**:1858-1869.
20. Barker G, Contreras JM, Heilman B, Singh AK, Verma RK, Nascimento M. Evolving Men: Initial Results from the International Men and Gender Equality Survey (IMAGES). Washington, D.C.: International Center for Research on Women (ICRW) and Instituto Promundo; 2011. Available: https://www.icrw.org/wp-content/uploads/2016/10/Evolving-Men-Initial-Results-from-the-International-Men-and-Gender-Equality-Survey-IMAGES-1.pdf. Accessed 16 Feb 2020
21. Gottert A, Pulerwitz J, Heck CJ, Cawood C, Mathur S. Creating HIV risk profiles for men in South Africa: a latent class approach using cross-sectional survey data. *J Int AIDS Soc* 2020; **23 (Suppl 2)**:e25518.
22. Shattuck D, Burke H, Ramirez C, Succop S, Costenbader B, Dekyem Attafuah J, *et al.* Using the Inequitable Gender Norms Scale and associated HIV risk behaviors among men at high risk for HIV in Ghana and Tanzania. *Men and Masculinities* 2013; **16**:540-559.
23. Shannon K, Leiter K, Phaladze N, Hlanze Z, Tsai AC, Heisler M, *et al.* Gender inequity norms are associated with increased male-perpetrated rape and sexual risks for HIV infection in Botswana and Swaziland. *PLoS One* 2012; **7**:e28739.
24. Fladseth K, Gafos M, Newell ML, McGrath N. The impact of gender norms on condom use among HIV-positive adults in KwaZulu-Natal, South Africa. *PLoS One* 2015; **10**:e0122671.
25. Dickson KS, Seidu AA, Eliason S, Darteh F, Darteh EKM. Intimate partner violence approval in South Africa: evidence from the 2016 Demographic and Health Survey. *Global Social Welfare* 2021; **8**:243-250.
26. Baranczuk Z, Estill J, Blough S, Meier S, Merzouki A, Maathuis MH, *et al.* Socio-behavioural characteristics and HIV: findings from a graphical modelling analysis of 29 sub-Saharan African countries. *J Int AIDS Soc* 2019; **22**:e25437.
27. Abrahams N, Jewkes R, Laubscher R, Hoffman M. Intimate partner violence: prevalence and risk factors for men in Cape Town, South Africa. *Violence Vict* 2006; **21**:247-264.
28. Rigby SW, Johnson LF. The relationship between intimate partner violence and HIV: a model-based evaluation. *Infectious Disease Modelling* 2017; **2**:71-89.
29. Pettifor A, Lippman SA, Gottert A, Suchindran CM, Selin A, Peacock D, *et al.* Community mobilization to modify harmful gender norms and reduce HIV risk: results from a community cluster randomized trial in South Africa. *J Int AIDS Soc* 2018; **21**:e25134.
30. Gibbs A, Washington L, Abdelatif N, Chirwa E, Willan S, Shai N, *et al.* Stepping Stones and Creating Futures intervention to prevent intimate partner violence among young people: cluster randomized controlled trial. *J Adolesc Health* 2020; **66**:323-335.
31. Wesson P, Lippman SA, Neilands TB, Twine R, Ahern J, Gomez-Olive FX, *et al.* Multilevel gender-equitable norms and risk of HIV and herpes simplex virus type 2 acquisition among young South African women: a longitudinal analysis of the HIV Prevention Trials Network 068 Cohort. *J Adolesc Health* 2019; **65**:730-737.

32. Jewkes R, Nduna M, Levin J, Jama N, Dunkle K, Puren A, *et al.* Impact of Stepping Stones on incidence of HIV and HSV-2 and sexual behaviour in rural South Africa: cluster randomised controlled trial. *BMJ* 2008; **337**:a506.
33. Saunders JB, Aasland OG, Babor TF, de la Fuente JR, Grant M. Development of the Alcohol Use Disorders Identification Test (AUDIT): WHO Collaborative Project on Early Detection of Persons with Harmful Alcohol Consumption - II. *Addiction* 1993; **88**:791-804.
34. Probst C, Shuper PA, Rehm J. Coverage of alcohol consumption by national surveys in South Africa. *Addiction* 2017; **112**:705-710.
35. Gordon AJ, Maisto SA, McNeil M, Kraemer KL, Conigliaro RL, Kelley ME, *et al.* Three questions can detect hazardous drinkers. *J Fam Pract* 2001; **50**:313-320.
36. Madhombiro M, Kidd M, Dube B, Dube M, Mutsvuke W, Muronzie T, *et al.* Effectiveness of a psychological intervention delivered by general nurses for alcohol use disorders in people living with HIV in Zimbabwe: a cluster randomized controlled trial. *J Int AIDS Soc* 2020; **23**:e25641.
37. Wandera B, Tumwesigye NM, Nankabirwa JI, Mafigiri DK, Parkes-Ratanshi RM, Kapiga S, *et al.* Efficacy of a single, brief alcohol reduction intervention among men and women living with HIV/AIDS and using alcohol in Kampala, Uganda: A randomized trial. *Journal of the International Association of Providers of AIDS Care* 2017; **16**:276-285.
38. Huis in 't Veld D, Ensoy-Musoro C, Pengpid S, Peltzer K, Colebunders R. The efficacy of a brief intervention to reduce alcohol use in persons with HIV in South Africa, a randomized clinical trial. *PLoS One* 2019; **14**:e0220799.
39. Vellios NG, van Walbeek CP. Self-reported alcohol use and binge drinking in South Africa: Evidence from the National Income Dynamics Study, 2014 – 2015. *S Afr Med J* 2018; **108**:33-39.
40. Reddy SP, James S, Sewpaul R, Koopman F, Funani NI, Sifunda S, *et al.* Umthente uhlaba usamila - The South African Youth Risk Behaviour Survey 2008. Cape Town: South African Medical Research Council; 2010.
41. Peltzer K, Davids A, Njuho P. Alcohol use and problem drinking in South Africa: findings from a national population-based survey. *African Journal of Psychiatry* 2011; **14**:30-37.
42. Papas RK, Gakinya BN, Mwaniki MM, Keter AK, Lee H, Loxley MP, *et al.* Associations between the phosphatidylethanol alcohol biomarker and self-reported alcohol use in a sample of HIV-infected outpatient drinkers in western Kenya. *Alcohol Clin Exp Res* 2016; **40**:1779-1787.
43. Bajunirwe F, Haberer JE, Boum Y, 2nd, Hunt P, Mocello R, Martin JN, *et al.* Comparison of self-reported alcohol consumption to phosphatidylethanol measurement among HIV-infected patients initiating antiretroviral treatment in southwestern Uganda. *PLoS One* 2014; **9**:e113152.
44. Adong J, Fatch R, Emenyonu NI, Cheng DM, Muyindike WR, Ngabirano C, *et al.* Social desirability bias impacts self-reported alcohol use among persons with HIV in Uganda. *Alcohol Clin Exp Res* 2019; **43**:2591-2598.
45. Francis JM, Weiss HA, Helander A, Kapiga SH, Changalucha J, Grosskurth H. Comparison of self-reported alcohol use with the alcohol biomarker phosphatidylethanol among young people in northern Tanzania. *Drug and Alcohol Dependence* 2015; **156**:289-296.
46. Boniface S, Scholes S, Shelton N, Connor J. Assessment of non-response bias in estimates of alcohol consumption: applying the continuum of resistance model in a general population Survey in England. *PLoS One* 2017; **12**:e0170892.

47. Stockwell T, Zhao J, Chikritzhs T, Greenfield TK. What did you drink yesterday? Public health relevance of a recent recall method used in the 2004 Australian National Drug Strategy Household Survey. *Addiction* 2008; **103**:919-928.
48. Parry CD, Pluddemann A, Steyn K, Bradshaw D, Norman R, Laubscher R. Alcohol use in South Africa: findings from the first Demographic and Health Survey (1998). *J Stud Alcohol* 2005; **66**:91-97.
49. Bello B, Moultrie H, Somji A, Chersich MF, Watts C, Delany-Moretlwe S. Alcohol use and sexual risk behaviour among men and women in inner-city Johannesburg, South Africa. *BMC Public Health* 2017; **17 (Suppl 3)**:548.
50. Nkosi S, Rich EP, Morojele NK. Alcohol use, sexual relationship power, and unprotected sex among patrons in bars and taverns in rural areas of North West province, South Africa. *AIDS Behav* 2014; **18**:2230-2239.
51. Pitpitan EV, Kalichman SC, Eaton LA, Cain D, Sikkema KJ, Watt MH*, et al.* Co-occurring psychosocial problems and HIV risk among women attending drinking venues in a South African township: a syndemic approach. *Annals of Behavioral Medicine* 2013; **45**:153-162.
52. Francis JM, Myers B, Nkosi S, Petersen Williams P, Carney T, Lombard C*, et al.* The prevalence of religiosity and association between religiosity and alcohol use, other drug use, and risky sexual behaviours among grade 8-10 learners in Western Cape, South Africa. *PLoS One* 2019; **14**:e0211322.
53. Weir SS, Pailman C, Mahlalela X, Coetzee N, Meidany F, Boerma JT. From people to places: focusing AIDS prevention efforts where it matters most. *AIDS* 2003; **17**:895-903.
54. Department of Health. South Africa Demographic and Health Survey 1998: Full Report. 1999.
55. Department of Health. South Africa Demographic and Health Survey 2003: Preliminary Report. Pretoria; 2004. Available: http://www.doh.gov.za/docs/reports/2003/sadhs2003/part2.pdf. Accessed 6 Jan 2012
56. Department of Health, Statistics South Africa, South African Medical Research Council, ICF. South Africa Demographic and Health Survey 2016. Pretoria; 2019. Available: https://www.dhsprogram.com/pubs/pdf/FR337/FR337.pdf. Accessed 19 March 2019
57. Shisana O, Rehle T, Simbayi LC, Parker W, Zuma K, Bhana A*, et al.* South African National HIV Prevalence, HIV Incidence, Behaviours and Communication Survey, 2005. Cape Town: HSRC Press; 2005. Available: http://www.hsrcpress.ac.za. Accessed 1 Dec 2005
58. Reddy SP, Panday S, Swart D, Jinabhai CC, Amosun SL, James S*, et al.* Umthenthe Uhlaba Usamila - The South African Youth Risk Behaviour Survey 2002. Cape Town: South African Medical Research Council; 2003. Available: http://www.mrc.ac.za/healthpromotion/healthpromotion.htm. Accessed 15 Dec 2003
59. Reddy P, James S, Sewpaul R, Sifunda S, Ellahebokus A, Kambaran NS*, et al.* Umthente Uhlaba Usamila – The 3rd South African National Youth Risk Behaviour Survey 2011. Cape Town: South African Medical Research Council; 2013. Available: https://africacheck.org/sites/default/files/3rd-Annual-Youth-Risk-Survey-2011.pdf. Accessed 22 July 2022
60. Cois A, Matzopoulos R, Pillay-van Wyk V, Bradshaw D. Bayesian modelling of population trends in alcohol consumption provides empirically based country estimates for South Africa. *Popul Health Metr* 2021; **19**:43.

61. Matzopolous R, Cois A, Probst C, Parry CDH, Vellios N, Sorsdahl K, *et al.* Estimating the changing burden of disease attributable to alcohol use in South Africa for 2000, 2006 and 2012. *S Afr Med J* 2022; **112**:662-675.
62. Stoebenau K, Heise L, Wamoyi J, Bobrova N. Revisiting the understanding of "transactional sex" in sub-Saharan Africa: A review and synthesis of the literature. *Soc Sci Med* 2016; **168**:186-197.
63. Jewkes R, Morrell R, Sikweyiya Y, Dunkle K, Penn-Kekana L. Transactional relationships and sex with a woman in prostitution: prevalence and patterns in a representative sample of South African men. *BMC Public Health* 2012; **12**:325.
64. Watt MH, Aunon FM, Skinner D, Sikkema KJ, Kalichman SC, Pieterse D. "Because he has bought for her, he wants to sleep with her": alcohol as a currency for sexual exchange in South African drinking venues. *Soc Sci Med* 2012; **74**:1005-1012.
65. Townsend L, Rosenthal SR, Parry CD, Zembe Y, Mathews C, Flisher AJ. Associations between alcohol misuse and risks for HIV infection among men who have multiple female sexual partners in Cape Town, South Africa. *AIDS Care* 2010; **22**:1544-1554.
66. Johnson LF, Mulongeni P, Marr A, Lane T. Age bias in survey sampling and implications for estimating HIV prevalence in men who have sex with men: insights from mathematical modelling. *Epidemiol Infect* 2018; **146**:1036-1042.
67. Mokgatle MM, Madiba S, Cele L. A comparative analysis of risky sexual behaviors, self-reported sexually transmitted infections, knowledge of symptoms and partner notification practices among male and female university students in Pretoria, South Africa. *International Journal of Environmental Research and Public Health* 2021; **18**:5660.
68. Dunkle KL, Jewkes RK, Brown HC, Gray GE, McIntryre JA, Harlow SD. Transactional sex among women in Soweto, South Africa: prevalence, risk factors and association with HIV infection. *Soc Sci Med* 2004; **59**:1581-1592.
69. Arnold MP, Struthers H, McIntyre J, Lane T. Contextual correlates of per partner unprotected anal intercourse rates among MSM in Soweto, South Africa. *AIDS Behav* 2013; **17 (Suppl 1)**:S4-11.
70. Adair T. Men’s Condom Use in Higher-Risk Sex: Trends and Determinants in Five Sub-Saharan Countries. *DHS Working Papers*; 2008. Available: https://www.dhsprogram.com/pubs/pdf/WP34/WP34.pdf. Accessed 3 Oct 2021
71. Pitpitan EV, Kalichman SC, Eaton LA, Watt MH, Sikkema KJ, Skinner D, *et al.* Men (and women) as "sellers" of sex in alcohol-serving venues in Cape Town, South Africa. *Prevention Science* 2014; **15**:296-308.
72. Magni S, Christofides N, Johnson S, Weiner R. Alcohol use and transactional sex among women in South Africa: results from a nationally representative survey. *PLoS One* 2015; **10**:e0145326.
73. Magni S, Hatcher A, Wamoyi J, Christofides N. Predictors and patterns of transactional sex with casual partners among adult men living in an informal urban area, South Africa. *AIDS Behav* 2020; **24**:2616-2623.
74. Dunkle KL, Jewkes R, Nduna M, Jama N, Levin J, Sikweyiya Y, *et al.* Transactional sex with casual and main partners among young South African men in the rural Eastern Cape: prevalence, predictors, and associations with gender-based violence. *Soc Sci Med* 2007; **65**:1235-1248.
75. Kalichman SC, Simbayi LC, Vermaak R, Cain D, Smith G, Mthebu J, *et al.* Randomized trial of a community-based alcohol-related HIV risk-reduction intervention for men and women in Cape Town South Africa. *Annals of Behavioral Medicine* 2008; **36**:270-279.

76. Wechsberg WM, Jewkes R, Novak SP, Kline T, Myers B, Browne FA, *et al.* A brief intervention for drug use, sexual risk behaviours and violence prevention with vulnerable women in South Africa: a randomised trial of the Women's Health CoOp. *BMJ Open* 2013; **3**.
77. Hatcher AM, Gibbs A, McBride RS, Rebombo D, Khumalo M, Christofides NJ. Gendered syndemic of intimate partner violence, alcohol misuse, and HIV risk among peri-urban, heterosexual men in South Africa. *Soc Sci Med* 2022; **295**:112637.
78. Duby Z, Jonas K, McClinton Appollis T, Maruping K, Vanleeuw L, Kuo C, *et al.* From survival to glamour: Motivations for engaging in transactional sex and relationships among adolescent girls and young women in South Africa. *AIDS Behav* 2021; **[In press]**.
79. Johnson LF, Hallett TB, Rehle TM, Dorrington RE. The effect of changes in condom usage and antiretroviral treatment coverage on HIV incidence in South Africa: a model-based analysis. *J Roy Soc Interface* 2012; **9**:1544-1554.
80. Dearing JW, Cox JG. Diffusion Of innovations theory, principles, and practice. *Health Affairs* 2018; **37**:183-190.
81. Berquo E, Barbosa RM, de Lima LP. Trends in condom use: Brazil 1998 and 2005. *Revista de Saúde Pública* 2008; **42 (Suppl 1)**:34-44.
82. Dinkelman T, Lam D, Leibbrandt M. Household and community income, economic shocks and risky sexual behavior of young adults: evidence from the Cape Area Panel Study 2002 and 2005. *AIDS* 2007; **21 (Suppl 7)**:S49-56.
83. Hargreaves JR, Morison LA, Kim JC, Busza J, Phetla G, Porter JD, *et al.* Characteristics of sexual partnerships, not just of individuals, are associated with condom use and recent HIV infection in rural South Africa. *AIDS Care* 2009; **21**:1058-1070.
84. Dunbar MS, Kang Dufour MS, Lambdin B, Mudekunye-Mahaka I, Nhamo D, Padian NS. The SHAZ! project: results from a pilot randomized trial of a structural intervention to prevent HIV among adolescent women in Zimbabwe. *PLoS One* 2014; **9**:e113621.
85. Kim J, Ferrari G, Abramsky T, Watts C, Hargreaves J, Morison L, *et al.* Assessing the incremental effects of combining economic and health interventions: the IMAGE study in South Africa. *Bull WHO* 2009; **87**:824-832.
86. Pettifor A, MacPhail C, Hughes JP, Selin A, Wang J, Gomez-Olive FX, *et al.* The effect of a conditional cash transfer on HIV incidence in young women in rural South Africa (HPTN 068): a phase 3, randomised controlled trial. *Lancet Glob Health* 2016; **4**:e978-e988.
87. Sikkema KJ, Watt MH, Meade CS, Ranby KW, Kalichman SC, Skinner D, *et al.* Mental health and HIV sexual risk behavior among patrons of alcohol serving venues in Cape Town, South Africa. *Journal of Acquired Immune Deficiency Syndrome* 2011; **57**:230-237.
88. Eaton LA, Cain DN, Pitpitan EV, Carey KB, Carey MP, Mehlomakulu V, *et al.* Exploring the relationships among food insecurity, alcohol use, and sexual risk taking among men and women living in South African townships. *J Prim Prev* 2014; **35**:255-265.
89. Gibbs A, Hatcher A, Jewkes R, Sikweyiya Y, Washington L, Dunkle K, *et al.* Associations between lifetime traumatic experiences and HIV-risk behaviors among young men living in informal settlements in South Africa: A cross-sectional analysis and structural equation model. *Journal of Acquired Immune Deficiency Syndrome* 2019; **81**:193-201.

90. Kalichman SC, Simbayi LC, Vermaak R, Cain D, Jooste S, Peltzer K. HIV/AIDS risk reduction counseling for alcohol using sexually transmitted infections clinic patients in Cape Town, South Africa. *Journal of Acquired Immune Deficiency Syndrome* 2007; **44**:594-600.
91. Papas RK, Gakinya BN, Mwaniki MM, Lee H, Keter AK, Martino S*, et al.* A randomized clinical trial of a group cognitive-behavioral therapy to reduce alcohol use among human immunodeficiency virus-infected outpatients in western Kenya. *Addiction* 2021; **116**:305-318.
92. L'Engle KL, Mwarogo P, Kingola N, Sinkele W, Weiner DH. A randomized controlled trial of a brief intervention to reduce alcohol use among female sex workers in Mombasa, Kenya. *Journal of Acquired Immune Deficiency Syndrome* 2014; **67**:446-453.
93. Kyegombe N, Abramsky T, Devries KM, Starmann E, Michau L, Nakuti J*, et al.* The impact of SASA!, a community mobilization intervention, on reported HIV-related risk behaviours and relationship dynamics in Kampala, Uganda. *J Int AIDS Soc* 2014; **17**:19232.
94. O'Connor EA, Perdue LA, Senger CA, Rushkin M, Patnode CD, Bean SI*, et al.* Screening and behavioral counseling interventions to reduce unhealthy alcohol use in adolescents and adults: updated evidence report and systematic review for the US Preventive Services Task Force. *JAMA* 2018; **320**:1910-1928.
95. Papas RK, Sidle JE, Gakinya BN, Baliddawa JB, Martino S, Mwaniki MM*, et al.* Treatment outcomes of a stage 1 cognitive-behavioral trial to reduce alcohol use among human immunodeficiency virus-infected out-patients in western Kenya. *Addiction* 2011; **106**:2156-2166.
96. McCambridge J. Reimagining brief interventions for alcohol: towards a paradigm fit for the twenty first century? *Addiction Science and Clinical Practice* 2021; **16**:41.
97. Fleming MF, Mundt MP, French MT, Manwell LB, Stauffacher EA, Barry KL. Brief physician advice for problem drinkers: long-term efficacy and benefit-cost analysis. *Alcoholism: Clinical and Experimental Research* 2002; **26**:36-43.
98. Conrod PJ, Castellanos-Ryan N, Mackie C. Long-term effects of a personality-targeted intervention to reduce alcohol use in adolescents. *Journal of Consulting and Clinical Psychology* 2011; **79**:296-306.
99. Wutzke SE, Conigrave KM, Saunders JB, Hall WD. The long-term effectiveness of brief interventions for unsafe alcohol consumption: a 10-year follow-up. *Addiction* 2002; **97**:665-675.
100. Dworkin SL, Treves-Kagan S, Lippman SA. Gender-transformative interventions to reduce HIV risks and violence with heterosexually-active men: a review of the global evidence. *AIDS Behav* 2013; **17**:2845-2863.
101. Ruane-McAteer E, Gillespie K, Amin A, Aventin A, Robinson M, Hanratty J*, et al.* Gender-transformative programming with men and boys to improve sexual and reproductive health and rights: a systematic review of intervention studies. *BMJ Global Health* 2020; **5**.
102. Levy JK, Darmstadt GL, Ashby C, Quandt M, Halsey E, Nagar A*, et al.* Characteristics of successful programmes targeting gender inequality and restrictive gender norms for the health and wellbeing of children, adolescents, and young adults: a systematic review. *Lancet Glob Health* 2020; **8**:e225-e236.
103. Muralidharan A, Fehringer F, Pappa S, Rottach E, Das M, Mandal M. Transforming Gender Norms, Roles, and Power Dynamics for Better Health: Evidence from a Systematic Review of Gender-integrated Health Programs in Low- and Middle-Income Countries. Washington, DC: Futures Group, Health Policy Project; 2015.

Available: https://www.healthpolicyproject.com/pubs/381_GPMIndiaSummaryReport.pdf. Accessed 15 April 2022
104. Press WH, Flannery BP, Teukolsky SA, Vetterling WT. Minimization or maximization of functions. In: *Numerical Recipes*. Cambridge: Cambridge University Press; 1986. pp. 274-334.
105. Smith AFM, Gelfand AE. Bayesian statistics without tears - a sampling resampling perspective. *Am Stat* 1992; **46**:84-88.
106. Staton CA, Vissoci JRN, El-Gabri D, Adewumi K, Concepcion T, Elliott SA, *et al.* Patient-level interventions to reduce alcohol-related harms in low- and middle-income countries: A systematic review and meta-summary. *PLoS Med* 2022; **19**:e1003961.
107. Scott-Sheldon LAJ, Carey KB, Johnson BT, Carey MP. Behavioural interventions targeting alcohol use among people living with HIV/AIDS: a systematic review and meta-analysis. *AIDS Behav* 2017; **21 (Suppl 2)**:126-143.
108. Burnhams NH, London L, Laubscher R, Nel E, Parry C. Results of a cluster randomised controlled trial to reduce risky use of alcohol, alcohol-related HIV risks and improve help-seeking behaviour among safety and security employees in the Western Cape, South Africa. *Substance Abuse Treatment, Prevention and Policy* 2015; **10**:18.
109. Chaudhury S, Brown FL, Kirk CM, Mukunzi S, Nyirandagijimana B, Mukandanga J, *et al.* Exploring the potential of a family-based prevention intervention to reduce alcohol use and violence within HIV-affected families in Rwanda. *AIDS Care* 2016; **28 (Suppl 2)**:118-129.
110. Rotheram-Borus MJ, Tomlinson M, Roux IL, Stein JA. Alcohol use, partner violence, and depression: a cluster randomized controlled trial among urban South African mothers over 3 years. *American Journal of Preventive Medicine* 2015; **49**:715-725.
111. Cubbins LA, Kasprzyk D, Montano D, Jordan LP, Woelk G. Alcohol use and abuse among rural Zimbabwean adults: a test of a community-level intervention. *Drug and Alcohol Dependence* 2012; **124**:333-339.
112. Wagman JA, Gray RH, Campbell JC, Thoma M, Ndyanabo A, Ssekasanvu J, *et al.* Effectiveness of an integrated intimate partner violence and HIV prevention intervention in Rakai, Uganda: analysis of an intervention in an existing cluster randomised cohort. *Lancet Glob Health* 2015; **3**:e23-33.
113. Pronyk PM, Hargreaves JR, Kim JC, Morison LA, Phetla G, Watts C, *et al.* Effect of a structural intervention for the prevention of intimate-partner violence and HIV in rural South Africa: a cluster randomised trial. *Lancet* 2006; **368**:1973-1983.
114. Mathews C, Eggers SM, Townsend L, Aaro LE, de Vries PJ, Mason-Jones AJ, *et al.* Effects of PREPARE, a multi-component, school-based HIV and intimate partner violence (IPV) prevention programme on adolescent sexual risk behaviour and IPV: cluster randomised controlled trial. *AIDS Behav* 2016; **20**:1821-1840.
115. Abramsky T, Devries K, Kiss L, Nakuti J, Kyegombe N, Starmann E, *et al.* Findings from the SASA! Study: a cluster randomized controlled trial to assess the impact of a community mobilization intervention to prevent violence against women and reduce HIV risk in Kampala, Uganda. *BMC Med* 2014; **12**:122.
116. Zule W, Myers B, Carney T, Novak SP, McCormick K, Wechsberg WM. Alcohol and drug use outcomes among vulnerable women living with HIV: results from the Western Cape Women's Health CoOp. *AIDS Care* 2014; **26**:1494-1499.
117. Shisana O, Rehle T, Simbayi LC, Zuma K, Jooste S, Pillay-van Wyk V, *et al.* South African national HIV prevalence, incidence, behaviour and communication survey,

2008: A turning tide among teenagers? Cape Town: Human Sciences Research Council; 2009. Available: http://www.hsrcpress.ac.za. Accessed 9 June 2009
118. Shisana O, Rehle T, Simbayi LC, Zuma K, Jooste S, Zungu N, *et al.* South African National HIV Prevalence, Incidence, and Behaviour Survey, 2012. Cape Town: Human Sciences Research Council; 2014. Available: http://www.hsrc.ac.za/en/research-outputs/view/6871. Accessed 16 April 2014
119. Simbayi LC, Zuma K, Zungu N, Moyo S, Marinda E, Jooste S, *et al.* South African National HIV Prevalence, Incidence, Behaviour and Communication Survey, 2017. Cape Town: Human Sciences Research Council; 2019. Available: https://www.hsrcpress.ac.za/books/south-african-national-hiv-prevalence-incidence-behaviour-and-communication-survey-2017. Accessed 6 Nov 2019
120. Bell GJ, Ncayiyana J, Sholomon A, Goel V, Zuma K, Emch M. Race, place, and HIV: The legacies of apartheid and racist policy in South Africa. *Soc Sci Med* 2022; **296**:114755.
121. Johnson LF, May MT, Dorrington RE, Cornell M, Boulle A, Egger M, *et al.* Estimating the impact of antiretroviral treatment on adult mortality trends in South Africa: a mathematical modelling study. *PLoS Med* 2017; **14**:e1002468.
122. Johnson LF, Keiser O, Fox MP, Tanser F, Cornell M, Hoffmann CJ, *et al.* Life expectancy trends in adults on antiretroviral treatment in South Africa. *AIDS* 2016; **30**:2545-2550.
123. Black A, Sitas F, Chibrawara T, Gill Z, Kubanje M, Williams B. HIV-attributable causes of death in the medical ward at the Chris Hani Baragwanath Hospital, South Africa. *PLoS One* 2019; **14**:e0215591.
124. Bor J, Chiu C, Ahmed S, Katz I, Fox MP, Rosen S, *et al.* Failure to initiate HIV treatment in patients with high CD4 counts: evidence from demographic surveillance in rural South Africa. *Trop Med Int Health* 2018; **23**:206-220.
125. Johnson LF, Dorrington RE. Thembisa version 4.5: A model for evaluating the impact of HIV/AIDS in South Africa. 2022. Available: https://www.thembisa.org/
